# Yap activation in irradiated parotid salivary glands is regulated by ROCK activity

**DOI:** 10.1371/journal.pone.0232921

**Published:** 2020-11-05

**Authors:** Wen Yu Wong, Kristy Gilman, Kirsten H. Limesand

**Affiliations:** 1 Cancer Biology Graduate Interdisciplinary Program, University of Arizona, Tucson, AZ, United States of America; 2 Department of Nutritional Sciences, University of Arizona, Tucson, AZ, United States of America; National Institute of Dental and Craniofacial Research, UNITED STATES

## Abstract

Radiotherapy plays a major role in the curative treatment of head and neck cancer, either as a single modality therapy, or in combination with surgery or chemotherapy, or both. Despite advances to limit radiation-induced side-effects, the major salivary glands are often affected. This frequently leads to hyposalivation which causes an increased risk for xerostomia, dental caries, mucositis, and malnutrition culminating in a significant impact on patients’ quality of life. Previous research demonstrated that loss of salivary function is associated with a decrease in polarity regulators and an increase in nuclear Yap localization in a putative stem and progenitor cell (SPC) population. Yap activation has been shown to be essential for regeneration in intestinal injury models; however, the highest levels of nuclear Yap are observed in irradiated salivary SPCs that do not regenerate the gland. Thus, elucidating the inputs that regulate nuclear Yap localization and determining the role that Yap plays within the entire tissue following radiation damage and during regeneration is critical. In this study, we demonstrate that radiation treatment increases nuclear Yap localization in acinar cells and Yap-regulated genes in parotid salivary tissues. Conversely, administration of insulin-like growth factor 1 (IGF1), known to restore salivary function in mouse models, reduces nuclear Yap localization and Yap transcriptional targets to levels similar to untreated tissues. Activation of Rho-associated protein kinase (ROCK) using calpeptin results in increased Yap-regulated genes in primary acinar cells while inhibition of ROCK activity (Y-27632) leads to decreased Yap transcriptional targets. These results suggest that Yap activity is dependent on ROCK activity and provides new mechanistic insights into the regulation of radiation-induced hyposalivation.

## Introduction

Radiotherapy has been an effective treatment option for patients with head and neck cancer. However, radiation treatment often generates severe side-effects that dramatically decrease patients’ quality of life. About two-thirds of long-term survivors whose salivary glands lie within the radiation field will experience irreversible hyposalivation and consequently other oral complications [1, 2]. Despite improvements in sparing the salivary glands through the use of intensity-modulated radiation therapy (IMRT), radiotherapy still leads to hyposalivation and other oral complications in a significant proportion of patients [3–5]. Current clinical management of this condition remains palliative and generally unsatisfactory.

Molecular mechanisms driving radiation-induced hyposalivation remain unclear. Some prevailing theories in the field include damaged stem and progenitor cell (SPC) pools, defective secretory pathways, irregular inflammatory responses, and cell death [4–9]. Recent studies have shown that the structural integrity of the epithelium is altered and that these defects are associated with aberrant apical-basolateral polarity, loss of lateral junctions and disorganized cytoskeletal structure [10–13]. In particular, radiation damage reduces the activity of an apical polarity protein, atypical protein kinase zeta (aPKCζ), in both SPCs and acinar cells resulting in hyperproliferation [11, 12]. Loss of aPKCζ in the SPCs promoted nuclear translocation of Yes-associated protein (Yap), a transcriptional co-activator regulated by Hippo signaling [11]. Interestingly, while nuclear Yap is essential for regeneration in a number of other injury models, the highest levels of nuclear Yap are observed in irradiated salivary glands that do not regenerate. Administration of insulin-like growth factor 1 (IGF1), known to restore salivary function in mouse models, led to a reduction in the number of SPCs exhibiting nuclear Yap [11]. However, following radiation damage or during tissue regeneration, the question of whether Yap localization is altered or what role it plays in these conditions in the entire tissue remains unknown.

Nuclear localization of Yap and its paralog Taz are known to promote proliferation, differentiation, stem cell fate, and organ size regulation [14, 15]. Taz has been shown to be redundant to Yap in most tissues; however distinct roles of Yap and Taz have been observed in the kidney and heart [16]. Deficiencies in Yap lead to developmental defects and hyperactivation of Yap promotes tumorigenesis [17]. Yap nuclear localization is broadly regulated by upstream regulators such as soluble mitogens, cell-cell contacts mediated by adherens junctions (AJs), and cytoskeletal regulation mediated in part by the extracellular matrix (ECM) [14, 15]. Genetic studies in *Drosophila* have identified the signaling cascade that regulates Yap. Upstream regulators activate Mst1/2 which in turn activates Lats1/2. Active Lats1/2 then phosphorylates Yap on serine 127 (pYap-S127) which induces formation of a complex with 14-3-3 thereby retaining Yap in the cytoplasm [14, 15]. In the absence of Hippo pathway activation, Yap translocates into the nucleus where it binds to one of the TEAD transcriptional enhancers to promote transcription of genes such as *Cyr61*, *Ccn2* and *Arhgef17* [14, 15, 17]. Alternatively, Yap phosphorylation and localization can be regulated by AJs and the cytoskeleton. Structural integrity and maintenance of junctional proteins are thought to bind and detain Yap/Taz at cellular junctions and thus suppress nuclear entry and activity [15, 18, 19]. It was recently shown in salivary glands that radiation damage decreases E-cadherin/β-catenin interactions and inhibition of a cytoskeletal regulator, Rho-associated coiled-coil kinase (ROCK), resulted in the restoration of the E-cadherin/β-catenin complex [20]. Thus, it is possible that alterations of ROCK or junctional proteins affect the stability of Yap/Taz following radiation damage and thus promote Yap-regulated activities.

In this study, we demonstrate that radiation damage induces Yap nuclear translocation and transcriptional activity but did not alter Taz levels. To determine whether alterations of ROCK activity induces nuclear Yap, primary cell cultures were treated with a ROCK activator, calpeptin. Treatment with calpeptin increased nuclear Yap localization. Conversely, inhibition of ROCK activity with Y-27632 in irradiated primary cell cultures prevented nuclear Yap translocation. ROCK inhibition in primary cell cultures mimicked similar phenotypes seen in an *in vivo* regeneration model where mice were treated with IGF1. These findings suggest that radiation-induced nuclear Yap localization is mediated by ROCK activity.

## Methods

### Mice and radiation treatment

Our prior studies have determined similar responses in both male and female animals [11, 21–23]; therefore, experiments were conducted in male and female FVB mice. Mice were maintained and treated in agreement with protocols approved by the University of Arizona Institutional Animal Care and Use Committee (IACUC). One dose of 5 Grey (5Gy) was administered with a ^60^Colbalt Teletherapy Instrument from Atomic Energy of Canada Ltd Theratron-80. The head and neck region was exposed while the rest of the body was shielded from radiation with >6mm thick lead to avoid systemic effects. Mice were anesthetized with an intraperitoneal injection of ketamine/xylazine (50mg/kg:10mg/mL) before radiation treatment and were monitored until they regained consciousness. Radiation dosage calculations and maintenance of the cobalt source were conducted by the Experimental Radiation Shared Service at the University of Arizona Cancer Center.

### Insulin-like Growth Factor 1 (IGF1) injections

Mice were given a maximum of four IGF-1 doses (5μg/mouse) via tail-vein injections 24-hours apart on days 4 to 7 following radiation treatment. For “Day 5” IGF1 treatments, mice received only one injection at day 4 post-irradiation and salivary glands were harvested 24 hours later. For “Day 30” IGF1 treatments, mice received four consecutive IGF1 treatments starting on day 4 post-irradiation and salivary glands were harvested on day 30 following radiation treatment.

### Immunoblotting

Whole protein lysates from parotid glands of FVB mice were harvested and processed for immunoblotting as previously described [11, 20]. Similarly, primary cell lysates were harvested and processed in the same fashion. Briefly, samples were lysed in RIPA buffer with 5mM sodium orthovanadate (Fisher Scientific, Hampton, NH), protease inhibitor cocktail (Sigma-Aldrich, St. Louis, MO), and 100mM PMSF (Thermo Scientific, Waltham, MA). The Coomassie Plus-The Better Bradford Assay (Thermo) was used to determine protein concentrations and 20-100ug total lysate was used. The following antibodies were used from Cell Signaling: anti-phospho-Yap (S127), anti-Yap, anti-Taz, anti-ERK1/2, anti-Lim domain kinase 2 (LIMK2), phosphorylated MLC, anti-MLC and anti-ROCK1. The total Yap antibody recognizes a domain on the carboxy terminus of the protein (region surrounding Pro435); therefore, it detects both phosphorylated and unphosphorylated proteins. Phosphorylated LIMK2 antibody was obtained from Thermo Scientific while pROCK antibody was obtained from Abcam. For detection, ECL substrate (Thermo Scientific) or SuperSignal West Pico Chemiluminescent Substrate (Thermo Scientific) was used. Restore Western Blotting Stripping Buffer (Fisher) was used to strip the membrane, reblocked with 2% BSA in 1X TBST and reprobed for loading controls.

### Immunofluorescent staining

Salivary glands were dissected at predetermined time points for formalin-fixed paraffin-embedded (FFPE) samples as previously described [11, 20]. Briefly, samples were fixed in formalin, embedded into paraffin and cut to 4um thickness by IDEXX BioResearch (Columbia, MO). Slides were rehydrated in graded ethanol, permeabilized in 0.02% Triton X-100, and antigen retrieval in 1mM citric acid buffer (pH 6.8). The slides were then blocked in 0.5% NEN (PerkinElmer, Waltham MA) and incubated in primary antibody overnight at 4°C. Secondary antibodies were added for 1 hour with Alexa Fluor 594 or 488 (Thermo Scientific). Samples were counterstained with DAPI (1ug/mL) and mounted with ProLong Gold Antifade Reagent (Life Technologies). Fluorescently stained slides were stored at 4°C for no longer than 5 days until imaging. The Yap antibody detects both phosphorylated and unphosphorylated forms and was purchased from Cell Signaling. Primary cells grown on collagen-coated glass coverslips (Neuvitro) were fixed with 3.7% formaldehyde, permeabilized with 0.1% Triton X-100 and stained with Alexa Fluor 488 Phalloidin (Invitrogen). Then, cells were counterstained with DAPI (1ug/mL) and mounted with ProLong Gold Antifade Reagent. Images were taken with a Leica DM5500 microscope (Leica Microsystems, Wetzlar, Germany) and a Spot Pursuit 4 Megapixel CCD camera (Diagnostic Instruments, Sterling Heights, MI) and Image J (NIH). Analysis of nuclear Yap cells was performed by manually counting positive cells from a minimum of 5–10 fields of view using a 40x objective. A minimum of three mice per group was analyzed. During analysis, we designated the ductal compartment based on morphological features, such as a rounded structure, the presence of a lumen, and tight cell-cell contacts. This compartment is primarily composed of excretory and striated ducts, as well as, some intercalated ducts. Thus, the acinar compartment comprises the remaining cells within salivary epithelium including acinar and myoepithelial cells, as well as, intercalated ducts that were not readily identifiable by morphology.

### Real-time RT-PCR

Parotid glands were removed from mice, stored in RNALater Stabilization Reagent (Qiagen, Valencia, CA) and processed as previously described [11, 20]. Briefly, RNA was isolated from parotid salivary glands with the RNeasy Mini Kit (Qiagen) and reversed transcribed with SuperScript IV Reverse Transcriptase (Invitrogen). Samples were analyzed in triplicate for each cDNA sample (3–5 mice per condition and at least 3 independent primary cell preps) with an iQ5 Real-Time PCR Detection System (Biorad). The data was analyzed using the 2^-ΔΔCT^ method. Results were normalized to *Gapdh*, which remains unchanged in response to treatment. Normalized values were graphed as relative fold-change compared to controls. The following primers were purchased from Integrated DNA Technologies (Coralville, IA): *Gapdh* (FWD: 5’-ACC ACA GTC CAT GCC ATC AC-3’; REV: 5’-CAC CAC CCT GTT GCT GTA GCC-3’); *Cyr61* (FWD: 5’- CTG CGC TAA ACA ACT CAA CGA-3’; REV: 5’- GCA GAT CCC TTT CAG AGC GG-3’) *Ccn2* (FWD: 5’ -CAG GAA GTA AGG GAC ACG AA 3’; REV: 5’–TGT GCG CTA ATG AAC AAC TG 3’) and *Arhgef17* (FWD: 5’–TAG CTT GAC ACA AAC TCG GT 3’; REV: 5’–GAG TGA AGG GTC AGC ATG TA 3’)

### Primary cell culture

Parotid glands were removed from euthanized mice and cultured as primary cells as previously described [20, 24]. Briefly, the glands from four mice were minced in dispersion media, mechanically agitated, washed, and seeded onto rat tail collagen plates or collagen-coated glass coverslips (Neuvitro) with primary cell culture media (Corning, Corning NY). For the ROCK activator experiments, cells were cultured with 10uM calpeptin, a ROCK activator, or DMSO vehicle control for 2 hours before RNA and protein were collected for downstream analyses. For cell irradiations, on day 1 after dissection, cells were exposed to a single dose of 5Gy radiation. For the ROCK inhibitor experiments, the cells were grown to sub-confluency and treated with either 20μM Y-27632 inhibitor (Calbiochem) or vehicle control on day 4 following radiation treatment. Cells were harvested on day 5 following radiation treatment and protein lysates and RNA were collected for downstream analysis.

### Statistics

Data were analyzed using Prism 6.04 (GraphPad, La Jolla, CA). All values were reported as the mean ± standard error of at least three independent experiments. No data points from individual mice or primary culture preparations were excluded from analysis. Student’s t-test was applied to results in which only two groups (vehicle vs. inhibitor) were compared. A one-way analysis of variance (ANOVA) test and a Tukey multiple comparison test was used to compare the results within different group means and considered significantly different at p<0.05. Treatment groups with different letters above the bar graphs are significantly different from each other (e.g. a treatment group denoted “A” is significantly different from a treatment group denoted “B” but not significantly different from another a treatment group denoted “A” or “AB”).

## Results

### Yap activation is increased in irradiated salivary glands

**Fig 1:**
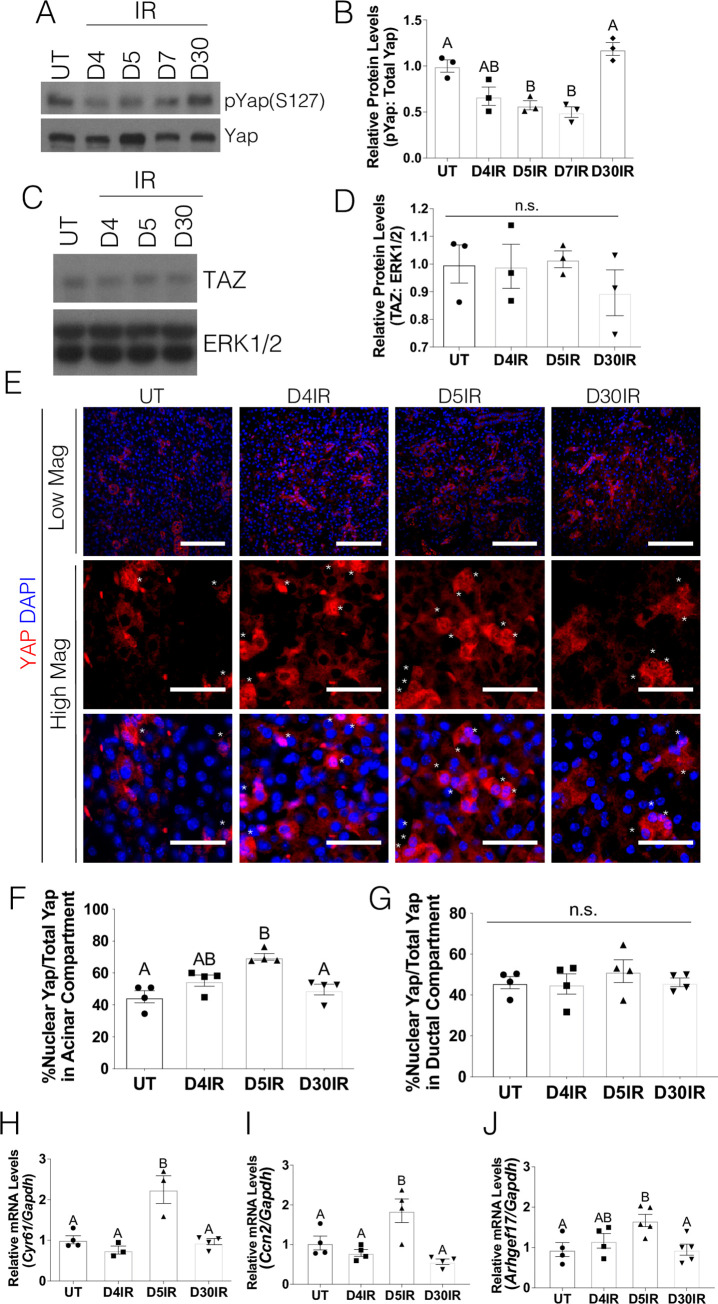
Yap activation is increased in irradiated salivary glands. FVB mice were either untreated (UT) or irradiated (IR) with 5Gy and dissected on days 4, 5, 7, and 30 following radiation treatment. Levels of (A-B) phosphorylated Yap on serine 127 (pYap S127) and (C-D) Taz were evaluated following radiation treatment. Immunoblots were re-probed with total Yap (A) or ERK (C) as a loading control. (E) Immunofluorescent staining was used to determine the percentage of cells that displayed nuclear Yap (red) in the acinar compartment (denoted by an asterisk). Composite images with DAPI (blue) are presented in both high and low magnification (scale bar for high magnification = 30 *μ*m, low magnification = 100 *μ*m). Quantification of the percentage of Yap positive cells with nuclear Yap staining from E was determined in the acinar enriched (F) and ductal (G) compartments separately. Relative mRNA levels of *Cyr61* (H), *Ccn2* (I) and *Arhgef17* (J) were determined by qRT-PCR and normalized to *Gapdh*. Results are presented from at least four mice per condition (each data point graphed in B, D, F-J represents individual mice); E- G used 10–15 images per mouse; error bars denote mean ± SEM. Multiple comparisons were conducted using a Tukey-Kramer test and significant differences (p<0.05) between treatment groups are denoted with different letters above the bar graphs. Treatment groups with different letters are significantly different from each other.

Prior work in a putative salivary SPC population revealed prolonged nuclear localization of Yap following radiation treatment [11]. In order to determine whether Yap is activated by radiation treatment across the entire salivary gland, we first examined the effects of a 5Gy dose on Yap phosphorylation. Given the partially compensatory role of Taz and evidence that Taz regulation can be distinct from Yap in some tissues [25, 26], total Taz protein levels were evaluated. Phosphorylation of Yap on serine 127 (S127) generates a 14-3-3 binding motif responsible for Yap cytoplasmic retention and prevents nuclear localization and transcriptional activity. In comparison to untreated mice, there is a decrease in pYap (S127) in irradiated mice on days 4, 5, and 7 (Fig 1A and 1B). On the other hand, Taz levels remain constant following radiation treatment (Fig 1C and 1D). Immunofluorescent staining for Yap using a total antibody that detects both phosphorylated and unphosphorylated proteins was performed to compare the percentage of cells that displayed nuclear Yap following radiation treatment. Cells within major ducts (excretory and striated ducts) have a distinctive response following radiation as evidenced by a lack of apoptosis at acute time points, a lack of induction of a compensatory proliferation response and their continued presence in clinical sections [11, 27–30]. Therefore, ductal cells were quantified separately and the remaining cellular populations were combined and labeled as the acinar compartment (Fig 1E–1G). In untreated mice, nuclear Yap is observed in approximately 40% of cells (asterisk denotes nuclear Yap staining in the acinar compartment). Comparatively, irradiated mice 5 days post-treatment display a higher percentage of nuclear Yap in the cell populations that comprise the acinar compartment which correlates with the time points where pYap (S127) is decreased (Fig 1A and 1B). In contrast to the acinar compartment, there was no change in the percentage cells with nuclear Yap in the cells that comprise the major ducts (Fig 1G). In comparison to untreated mice, irradiated mice display significantly elevated mRNA transcripts for Yap transcriptional targets, *Cyr61*, *Ccn2* and *Arhgef17*, (Fig 1H–1J) five days after treatment. By 30 days post-radiation, Yap activation appears to return to baseline levels as indicated by Yap phosphorylation, nuclear localization and transcript levels of *Cyr61 Ccn2* and *Arhgef17*. These results suggest that radiation activates Yap in the salivary acinar compartment during a window 5–7 days after radiation.

### Post-therapeutic IGF1 treatment reduces Yap activation

**Fig 2:**
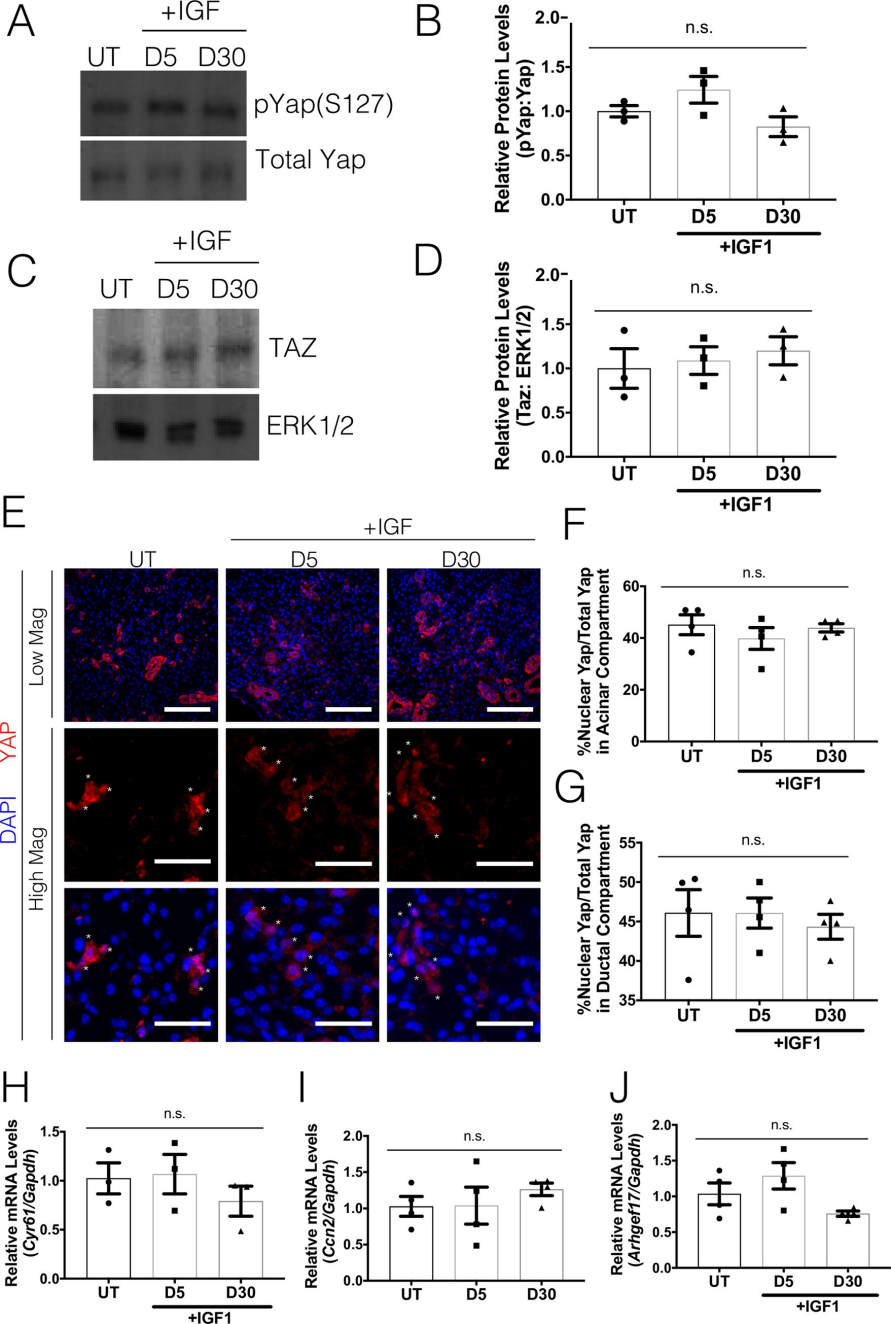
Post-therapeutic IGF1 treatment reduces Yap activation. FVB mice (4–6 weeks old) were either untreated (UT) or irradiated (IR) with 5Gy and given post-therapeutic IGF1 (IR+IGF) on days 4–7. Parotid salivary glands were dissected on days 5 and 30 following radiation treatment. Levels of (A-B) phosphorylated Yap on serine 127 (pYap S127) and (C-D) Taz were evaluated following IR+IGF treatment. Immunoblots were re-probed with total Yap or ERK as a loading control. (E) Immunofluorescent staining was used to determine the percentage of cells that displayed nuclear Yap (red) in the acinar compartment. Composite images with DAPI (blue) are presented in both high and low magnification (scale bar for high magnification = 30 μm, low magnification = 100 μm). Quantification of the percentage of Yap positive cells with nuclear Yap staining from E was determined in the acinar enriched (F) and ductal (G) compartments separately. Relative mRNA levels of *Cyr61* (H), *Ccn2* (I) and *Arhgef17* (J) were determined by qRT-PCR and normalized to *Gapdh*. Results are presented from at least four mice per condition (each data point graphed in B, D, F*-*J represents individual mice); E-F used 10–15 images per mouse; error bars denote mean ± SEM. Multiple comparisons were conducted using a Tukey-Kramer test and significant differences (p<0.05) between treatment groups are denoted with different letters above the bar graphs. Treatment groups with different letters are significantly different from each other.

Previous studies have shown that systemic post-therapeutic administration of IGF1 restores salivary function in terms of apical-basolateral polarity, salivary secretion and production of essential salivary proteins by day 30 post-irradiation [11, 28]. However, it is currently unclear how IGF1 administration restores salivary function and whether Yap activation is impacted during the regenerative process. Therefore, Yap activation was analyzed in parotid glands at days 5 and 30 post-radiation in mice receiving one or four injections of IGF1 respectively. In comparison to untreated mice, IGF1 treated mice displayed similar levels of phosphorylated Yap on serine 127 (pYap-S127) as early as day 5 and maintained as late as day 30 post-irradiation (Fig 2A and 2B). Similar to irradiated salivary glands, mice treated with IGF1 post-radiation displayed comparable levels of Taz across both time points (Fig 2C and 2D). Immunofluorescent staining for Yap was performed to compare the percentage of cells that displayed nuclear Yap in the acinar or ductal compartments (Fig 2E–2G). Comparatively, irradiated mice that received IGF1 have similar levels of Yap nuclear localization as untreated salivary glands. Yap transcriptional target genes, *Cyr61*, *Ccn2* and *Arhgef17* (Fig 2H–2J), are also present at similar levels between untreated and IGF1 treated mice at both time points. These results indicate that IGF1 treatment is able to reduce radiation-induced Yap activation to levels comparable to unirradiated controls.

### Activation of ROCK leads to Yap transcriptional activity

**Fig 3:**
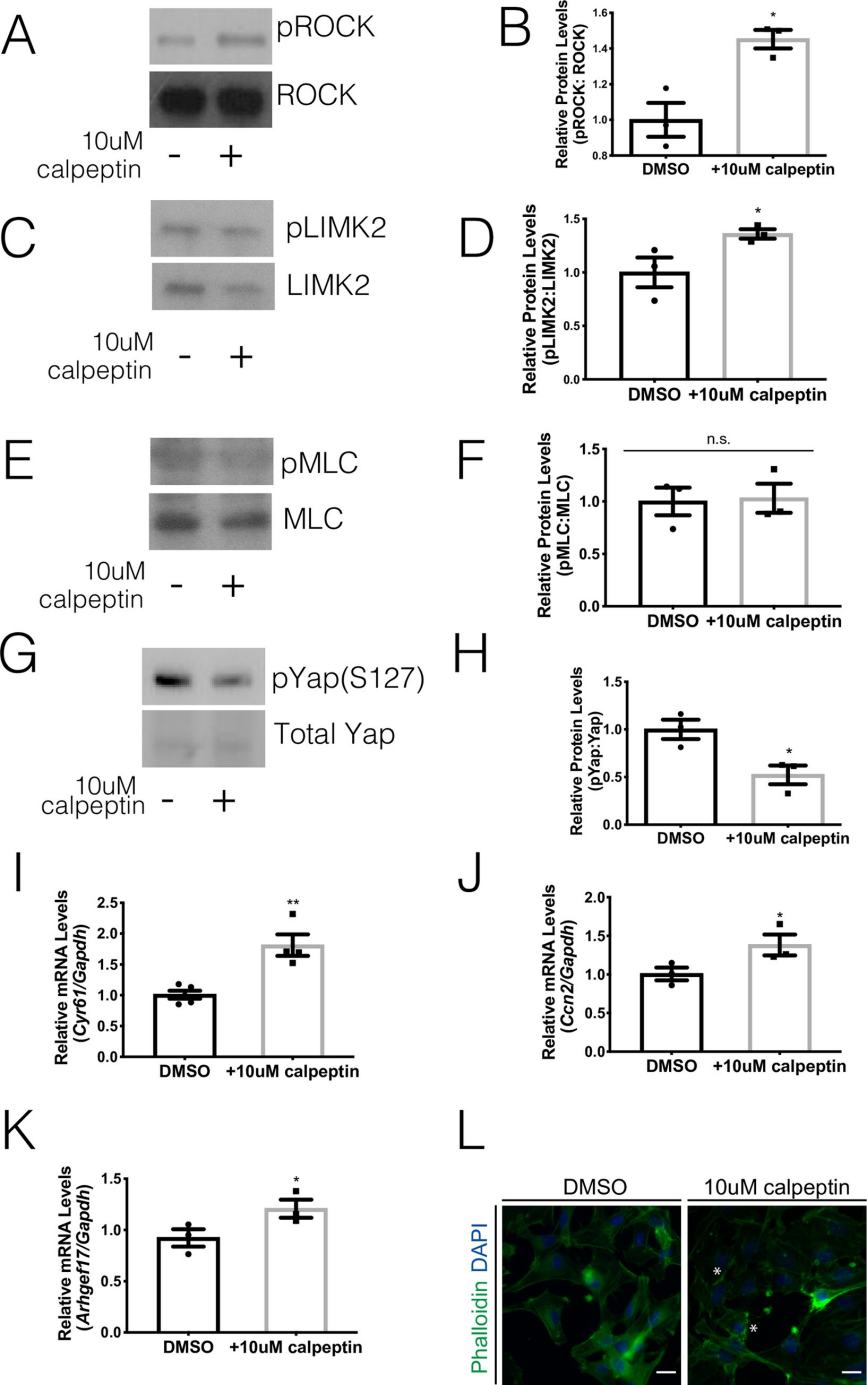
Activation of ROCK leads to Yap transcriptional activity. Parotid salivary glands from FVB mice were dissected and cultured as primary acinar cell cultures. At sub-confluency, the cells were treated 10 μM calpeptin (ROCK activator) or DMSO vehicle control for two hours. (A) Levels of phosphorylated ROCK were determined by immunoblot following calpeptin treatment. Immunoblots were reprobed for total ROCK as a loading control. (B) Quantification of A. (C) Levels of phosphorylated LIMK2 were determined by immunoblot following calpeptin treatment. Immunoblots were reprobed for total LIMK2 as a loading control. (D) Quantification of C. (E) Levels of phosphorylated MLC were determined by immunoblot following calpeptin treatment. Immunoblots were reprobed for total MLC as a loading control. (F) Quantification of E. (G) Levels of phosphorylated Yap on serine 127 (pYAP S127) were determined following calpeptin treatment. Immunoblots were reprobed for total Yap as a loading control. (H) Quantification of G. (E) Relative mRNA levels of *Cyr61* (I), *Ccn2* (J) and *Arhgef17* (K) were determined by qRT-PCR and normalized to *Gapdh*. (L) Immunofluorescent staining with Phalloidin (green) was used to visualize F-actin structures after calpeptin treatment. Indications of F-actin fragmentation are noted with asterisks (scale bar for magnification = 20 μm). Results are presented from at least three independent *in vitro* primary cell culture experiments per condition (each data point graphed in B, D, F, and H-K represents an independent cell preparation); error bars denote mean ± SEM. Significant difference (p<0.05) between vehicle control and inhibitor treated cells was determined by Student’s t-test.

Previous research in the skin demonstrated that changes in cytoskeletal regulators could influence Yap localization [31, 32]. One key cytoskeletal regulator is ROCK, a protein that is often associated with junctional complexes and maintains the integrity of the epithelium [33]. Our previous work uncovered that radiation treatment of salivary glands leads to elevated ROCK signaling on Day 5 *in vivo* leading to phosphorylation of downstream targets (LIMK2 and MLC) and fragmentation of actin filaments [20]. This increase in ROCK signaling corresponds to a time point when there is an increase in Yap activation (Fig 1). To determine whether ROCK signaling could alter Yap activation, primary parotid cell cultures were treated with a ROCK activator, 10 μM calpeptin, or vehicle (DMSO). Levels of phosphorylated ROCK are elevated in cells treated with calpeptin, which leads to increases in phosphorylated LIMK2 (Fig 3A–3D). Phosphorylation of another ROCK downstream target, MLC, remains unchanged with calpeptin treatment potentially due to the acute time point evaluated (Fig 3E and 3F). Primary cells treated with calpeptin have lower levels of phosphorylated Yap on serine 127 (pYap-S127) than vehicle treated cells (Fig 3G and 3H). Concurrently, cells treated with calpeptin displayed elevated mRNA levels for Yap-target genes, *Cyr61*, *Ccn2* and *Arhgef17*, in comparison to DMSO treated cells (Fig 3I–3K). Treatment with calpeptin also appears to decrease F-actin levels with early indications of fragmentation (Fig 3L; noted with asterisks). These results suggest that ROCK activation in parotid primary cells stimulates Yap transcriptional activity.

### Inhibition of ROCK activity reduces Yap transcriptional activity following radiation treatment

**Fig 4:**
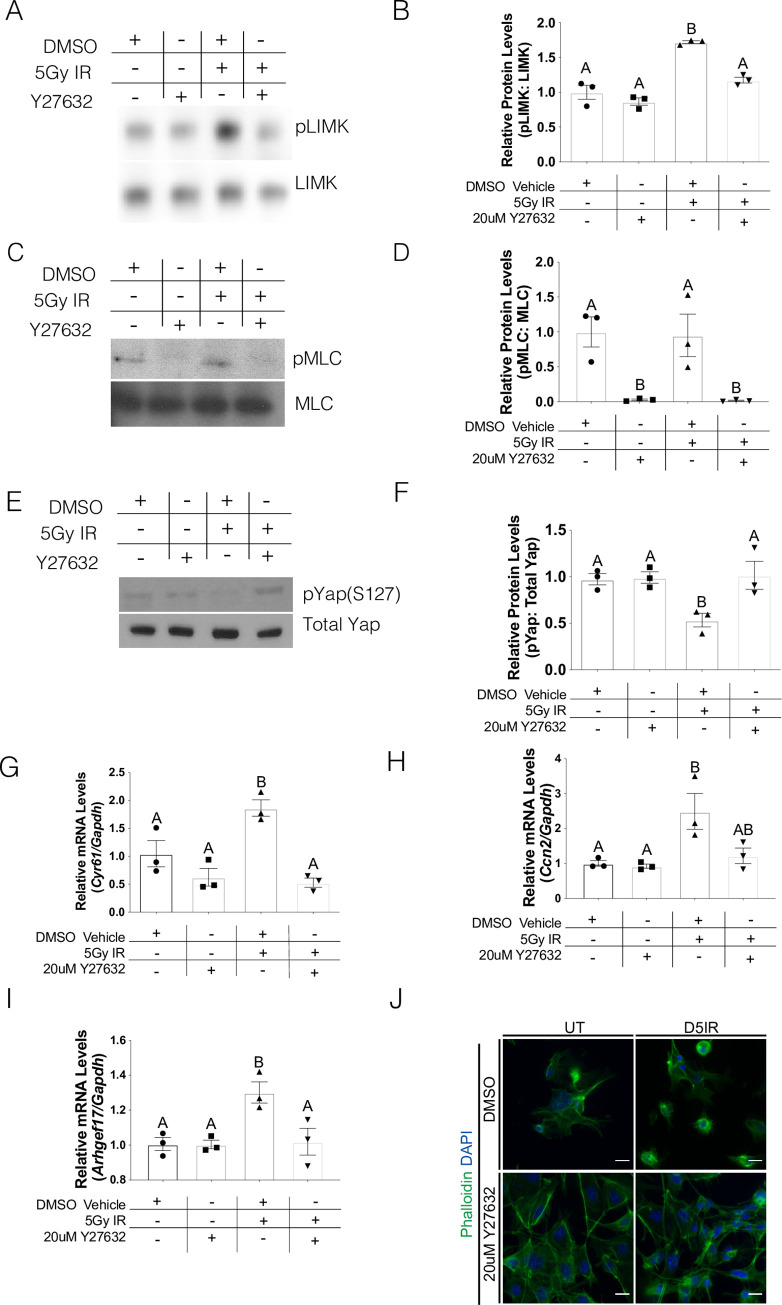
Inhibition of ROCK activity reduces Yap transcriptional activity following radiation treatment. Parotid salivary glands from FVB mice were dissected and cultured as primary acinar cell cultures. One day after dissection, the primary cells were irradiated with 5Gy and cell lysates or RNA were collected on Day 5 after radiation treatment. On Day 4 after radiation treatment, the cells were either treated with 20 μM Y-27632 (ROCK inhibitor) or vehicle control. Effects of ROCK inhibition on phosphorylated LIMK (A) or phosphorylated MLC (C) in primary salivary cells were evaluated by immunoblotting. Blots were reprobed for total levels of LIMK or MLC. (B) Quantification by densitometry of A normalized to vehicle control. (D) Quantification by densitometry of C normalized to vehicle control. (E) Effects of ROCK inhibition on phosphorylated Yap on serine 127 (pYap S127) were evaluated by immunoblotting. Blots were reprobed for total levels of Yap. (F) Quantification by densitometry of E normalized to vehicle control. (E) Relative mRNA levels of *Cyr61* (G), *Ccn2* (H) and *Arhgef17* (I) were determined by qRT-PCR and normalized to *Gapdh*. (J) Immunofluorescent staining with Phalloidin (green) was used to visualize F-actin structures after radiation with and without Y-27632 treatment (scale bar for magnification = 20 μm). Results are presented from at least three independent *in vitro* primary cell culture experiments per condition (each data point graphed in B, D, and F-I represents an independent cell preparation); error bars denote mean ± SEM. Multiple comparisons were conducted using a Tukey-Kramer test and significant differences (p<0.05) between treatment groups are denoted with different letters above the bar graphs. Treatment groups with different letters are significantly different from each other.

Our prior work on the ROCK signaling pathway following irradiation of salivary glands demonstrated that inhibition of ROCK activity led to the re-establishment of β-catenin/E-cadherin binding at lateral junctions [20]. To further understand the ability of ROCK signaling to impact Yap transcriptional activity, we evaluated whether a ROCK pharmacological inhibitor could alter Yap phosphorylation and levels of Yap target genes following radiation. Primary cell cultures were treated with either 20 μM Y-27632 or vehicle control on day 4 following radiation treatment and cells were collected 24 hours later (day 5 after radiation). Levels of ROCK substrates (pLIMK2 and pMLC) are reduced in cells treated with Y-27632, thereby confirming pathway inhibition with this compound (Fig 4A–4D). Irradiated cells treated with Y-27632 have similar levels of pYap-S127 as unirradiated cells, which is significantly higher than cells receiving radiation+vehicle (Fig 4E and 4F). Similarly, irradiated cells treated with Y-27632 had lower levels of Yap-target genes, *Cyr61*, *Ccn2* and *Arhgef17*, in comparison to irradiated cells (Fig 4G–4I). Strikingly, cells treated with Y-27632 have restored actin filament structure when compared to irradiated cells (Fig 4J). These results suggest that radiation-induced Yap transcriptional activity can be regulated by ROCK signaling.

## Discussion

Hyposalivation and xerostomia are common complications of collateral damage to salivary glands due to radiotherapy treatments for head and neck cancer. Radiation significantly alters salivary gland structure and differentiation leading to a state of chronic dysfunction. In the clinic, patients receive a fractionated radiation regime with the goal of limiting salivary gland exposure to 1.8–2.0 Gy/day [5, 29]. This study utilized a single dose of 5Gy, which is similar to the daily clinical fraction and sufficient to induce chronic salivary hypofunction [22, 27, 28, 30], in order to investigate the kinetic events that underscore the damaged phenotype. In the mouse model utilized in this study, apoptosis peaks at 24–48 hours post-treatment leading to significant decreases in stimulated salivary flow rates as early as three days post-treatment [22, 27, 30]. Decreased levels of apoptosis following radiation correlates with preserved stimulated salivary flow rates [27, 30]. Initiation of compensatory proliferation is observed five days post-treatment and proliferation levels remain increased chronically (≥90 days post-treatment) [11, 12, 28]. Injections of IGF1 post-radiation (days 4–7) decrease proliferation rates to untreated levels and restores stimulated salivary gland function to equivalent levels as unirradiated mice [28]. While increases in compensatory proliferation are a necessary step in wound healing, chronically elevated levels of proliferation can inhibit differentiation [34]. Therefore, understanding the molecular regulators in the induction and maintenance of compensatory proliferation is a critical step toward uncovering therapeutics that can restore salivary gland function in head and neck cancer patients that have completed radiotherapy.

Yap is known to mediate proliferation, differentiation, cell fate, and migration–all components needed for tissue restoration following damage [14, 17, 18, 31, 32]. Yap phosphorylation and elevated nuclear Yap localization in the stem cell compartment have been documented in epithelial injury and regeneration models such as the lung, skin, cornea and intestines [32, 35, 36]. However, we demonstrate that in salivary glands, there is a higher percentage of nuclear Yap and Yap-target genes throughout irradiated glands, beyond the SPC compartment (Fig 1). Interestingly, Yap activation in the entire salivary gland is present during a specific window (days 5–7) after radiation, while increased Yap nuclear translocation in a heterogeneous salivary progenitor population (LRC) occurred at day 5 until at least day 30 [11]. Post-radiation treatment with IGF1, an intervention known to restore salivary function, reduced nuclear Yap translocation in cells within the acinar compartment across the entire salivary gland (Fig 2) similar to previous results within the SPC compartment [11]. These differences between the SPC compartment and the entire salivary gland in nuclear Yap kinetics following radiation suggests a large portion of the salivary gland initially responds to extracellular cues that lead to Yap activation. In contrast, sustained translocation of Yap in the SPC compartment may be the result of additional signals that could indicate an altered wound healing response.

A number of transcriptional target genes have been shown to be induced following nuclear Yap translocation with involvement in cell growth, cell proliferation, cell plasticity, EMT, and cell migration [37–39]. The Yap transcriptional targets evaluated in each figure were selected as surrogates of Yap activation as well as having a connection to an *in vivo* irradiated salivary gland phenotype. *Cyr61* has been shown to play a role in cell proliferation, cell adhesion, chemotaxis and angiogenesis [40, 41]. These functional roles could impact the induction and regulation of compensatory proliferation and the loss of E-cadherin/β-catenin binding at adherns junctions in irradiated salivary gland tissue [12, 20, 28]. Increased levels of *Cyr61* have been shown at sites of inflammation and tissue repair with sustained increases described in a number of chronic inflammatory diseases [40]. CTGF (connective tissue growth factor, *Ccn2* gene) is a matricellular protein that plays a role in cell growth, cell proliferation and cell adhesion [40, 42]. Elevated levels of CTGF have been demonstrated to result in an accumulation of ECM during the development of fibrosis [43]. CTGF treatment of lung epithelial cells induced EMT [43], suggesting CTGF could affect the differentiation program following injury. Considerably less has been reported on *Arhgef17* function; however, as a RhoGEF family member it appears to have a role in actin filament binding activity [44]. This functional role would connect to previously reported alterations in F-actin organization in irradiated salivary glands [20]. The induction of these target genes within a specific window (days 5–7) after radiation suggests involvement in the cellular changes that occur with initiation of proliferation. Targeting Yap downstream effectors as a strategy to regulate cancer progression has been characterized as largely underdeveloped [45]; therefore, considerable work remains to determine the efficacy of this approach for treating irradiated salivary glands.

Proper regulation of the cytoskeletal and polarity mediator, ROCK, is essential for salivary gland development, enhanced salivary sphere formation, and prevention of altered structural integrity of the salivary epithelium [20, 46, 47]. It was previously demonstrated that radiation activates the ROCK signaling cascade leading to the dissociation of the E-cadherin/β-catenin complex and disruption of cytoskeletal structure [20]. Here, we demonstrate that upregulation of ROCK activity also upregulates Yap transcriptional activity (Fig 3), while inhibition of ROCK signaling prevents this activation following radiation damage (Fig 4). Mechanistically, upregulation of ROCK activity has been demonstrated to lie downstream of altered cell-ECM structure and/or chronic inflammation. Radiation damage is known to alter the cell-ECM landscape and alterations of these regulators have been shown to upregulate ROCK and metalloprotease activity, migration, and carcinogenesis [48–50]. In the cornea, upregulation of inflammatory cytokines results in excessive ECM deposition, upregulation of ROCK signaling and promotion of nuclear Yap/Taz translocation [36]. Upregulation of Yap target genes involved in ECM deposition (e.g. *Ccn2*, Fig 1) following radiation could represent a positive feedback loop leading to prolonged ROCK pathway activation and cytoskeletal disruptions previously described in irradiated salivary glands [20]. In addition, we and others have demonstrated that following radiation damage, there is elevated inflammatory molecules like prostaglandins (PGE_2_) and TNF-α that could potentially promote ROCK activity [7, 51]. Further studies are needed to understand the interplay of cell-cell interactions, inflammation and ROCK activation in radiation-induced nuclear Yap translocation.

The coordination of multiple signaling pathways and morphogens during salivary gland wound healing after radiation exposure is essential to guarantee robustness in the restoration of salivary function. Previous reports evaluating the initial period of compensatory proliferation in irradiated salivary glands established loss of E-cadherin/β-catenin binding, increased ROCK signaling, disrupted cytoskeletal network, loss of apical/basolateral polarity and increased JNK signaling [12, 20]. This work extends these observations to the ability of increased ROCK signaling in the regulation of nuclear Yap localization and activity during this same period. Importantly, administration of IGF1 post-radiation reverses all of these phenotypes [12, 20] as the tissue progresses to restoration of physiological function [28]. These results suggest targeting ROCK or Yap activation may have utility in patients that have recently completed radiotherapy. Clinical evaluation of ROCK inhibition appears to have focused on disorders of the eye (corneal wound healing and diabetic macular oedema) [52–54] and it is unclear whether the safety profile in these studies would translate to salivary glands. Inhibiting Yap activation has focused on modulating upstream regulators (e.g. mechanical signals) or disrupting binding to transcription factors or enhancers (e.g. TEAD) [45]. Careful consideration of the necessary role Yap plays in regeneration in other tissues [16, 55] needs to be balanced with the potential delay in wound healing progression in cellular populations of irradiated salivary glands. Therefore, further understanding of the distinctive roles that Yap plays during radiation damage and tissue regeneration may provide insights into the temporal manner in which nuclear Yap regulates tissue homeostasis.

## Supporting information

S1 File (PDF)
