# Supplementary material for: Yap activation in irradiated parotid salivary glands is regulated by ROCK activity
Source: PLoS One. 2020 Nov 5;15(11):e0232921. doi: 10.1371/journal.pone.0232921 (PMC7644026; doi:10.1371/journal.pone.0232921)
Supplement: S1 File — (PDF) [file pone.0232921.s001.pdf]

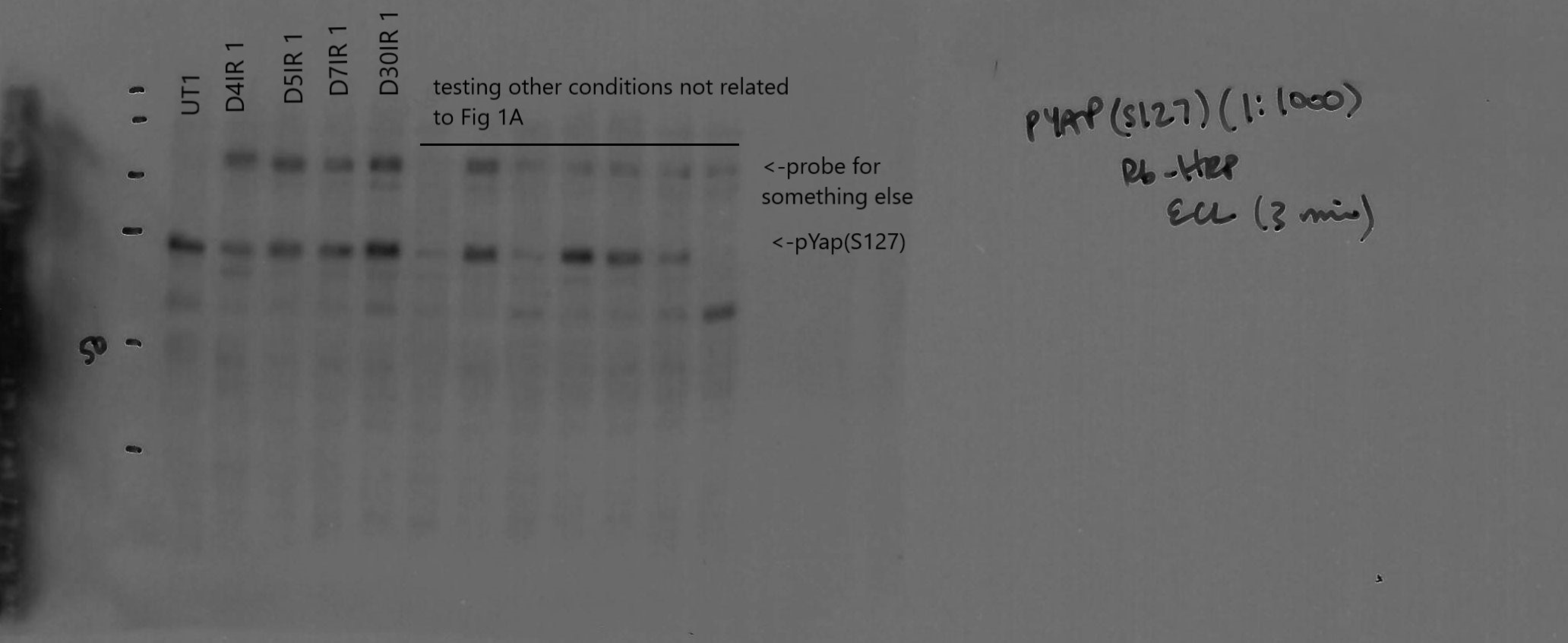
UT1
D4IR 1
D5IR 1
D7IR 1
D30IR 1
testing other conditions not related to Fig 1A
<-probe for something else
<-pYap(S127)
50
pYAP (S127) (1:1000)
Rb-HRP
ECL (3 min)

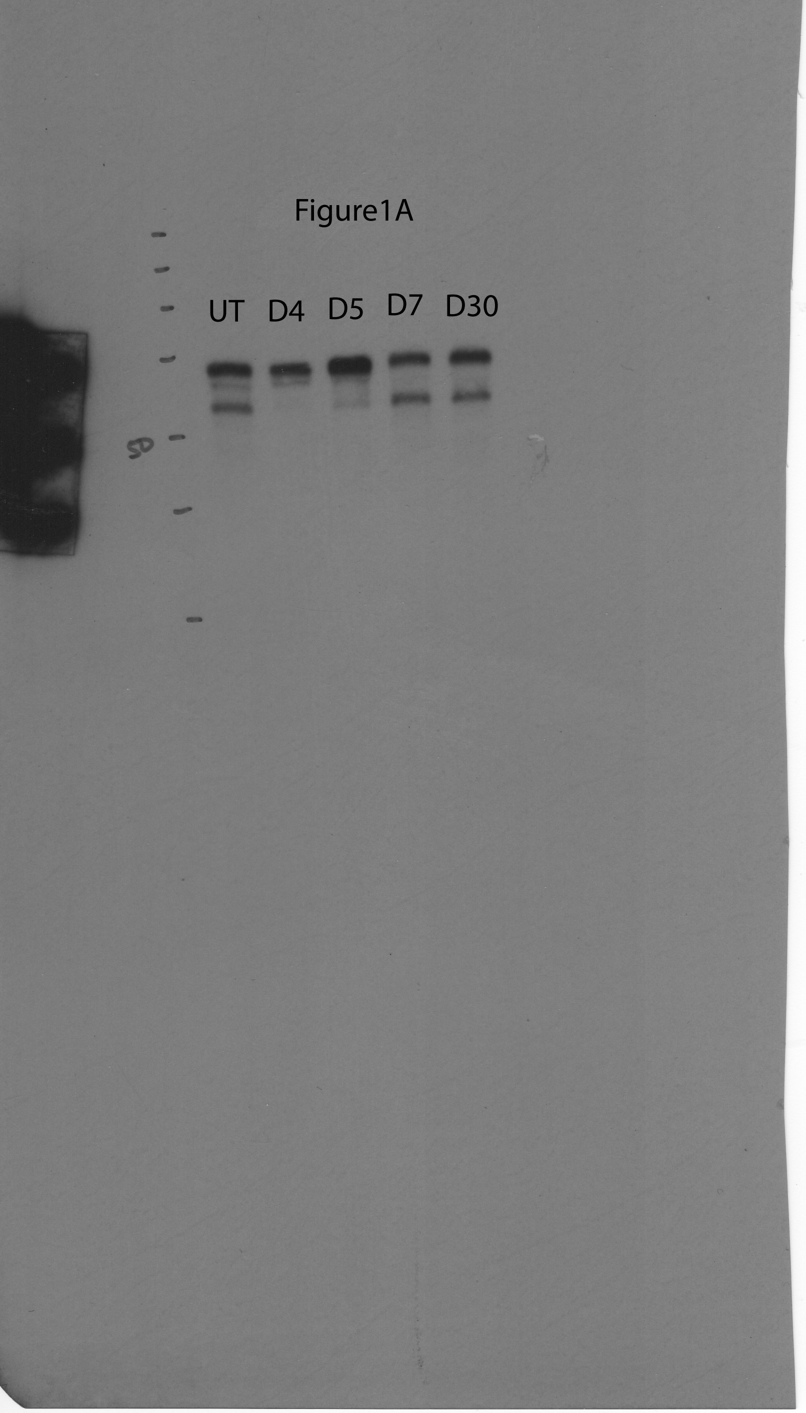

Figure1A

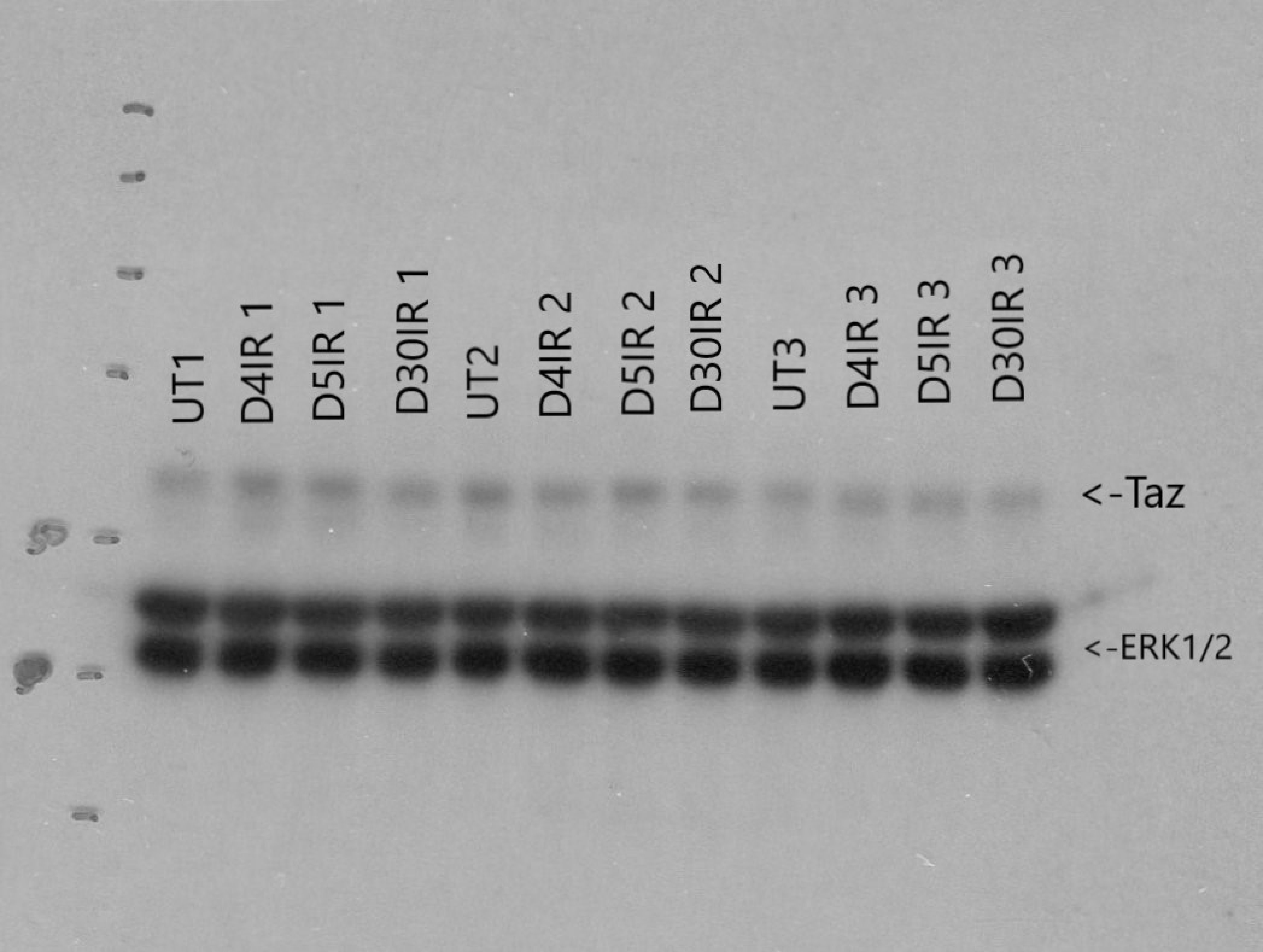
UT1
D4IR 1
D5IR 1
D30IR 1
UT2
D4IR 2
D5IR 2
D30IR 2
UT3
D4IR 3
D5IR 3
D30IR 3
<-Taz
<-ERK1/2

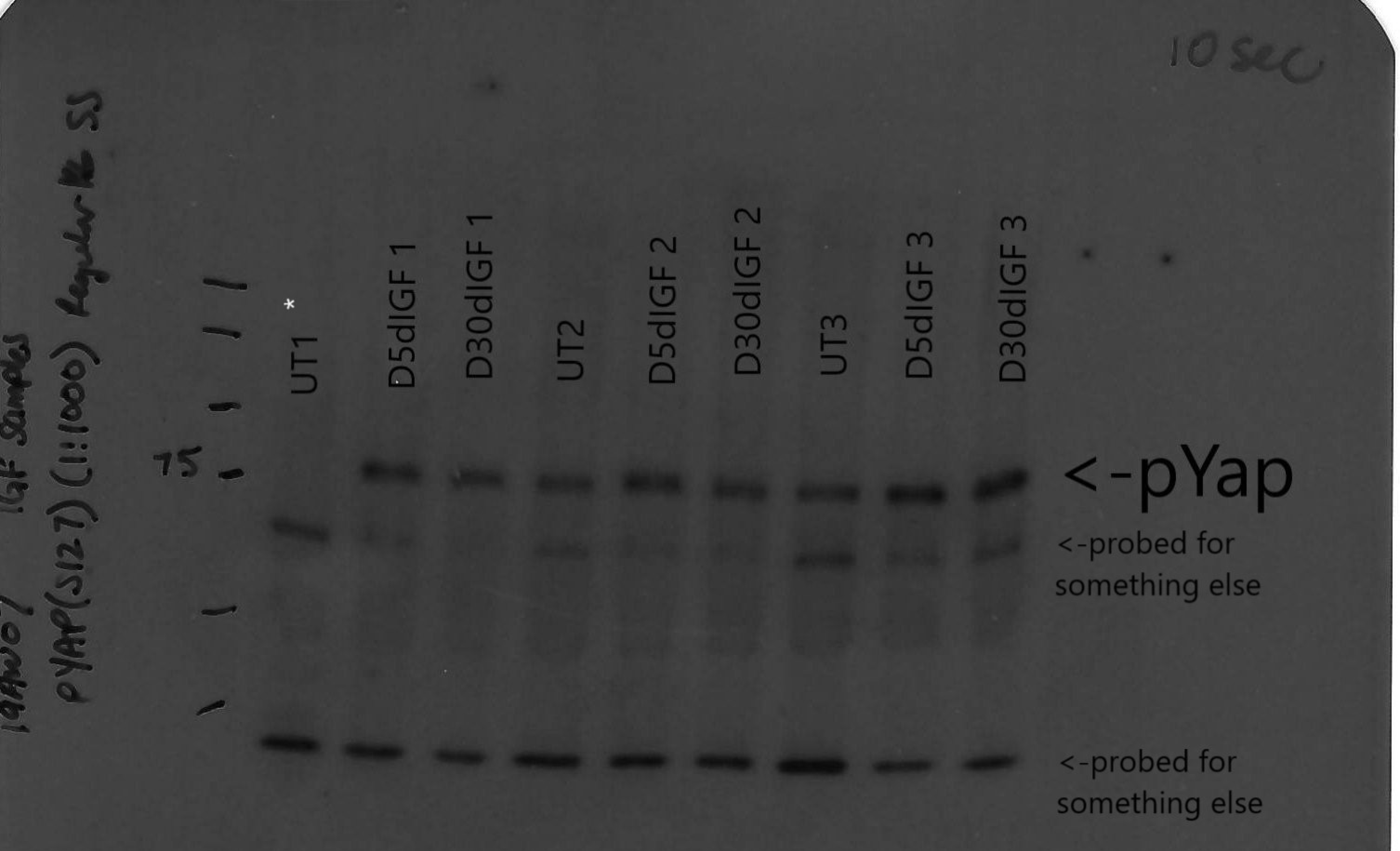

*Note: It looks like there's no pYap for UT1 but if you view blot at 1min it's there

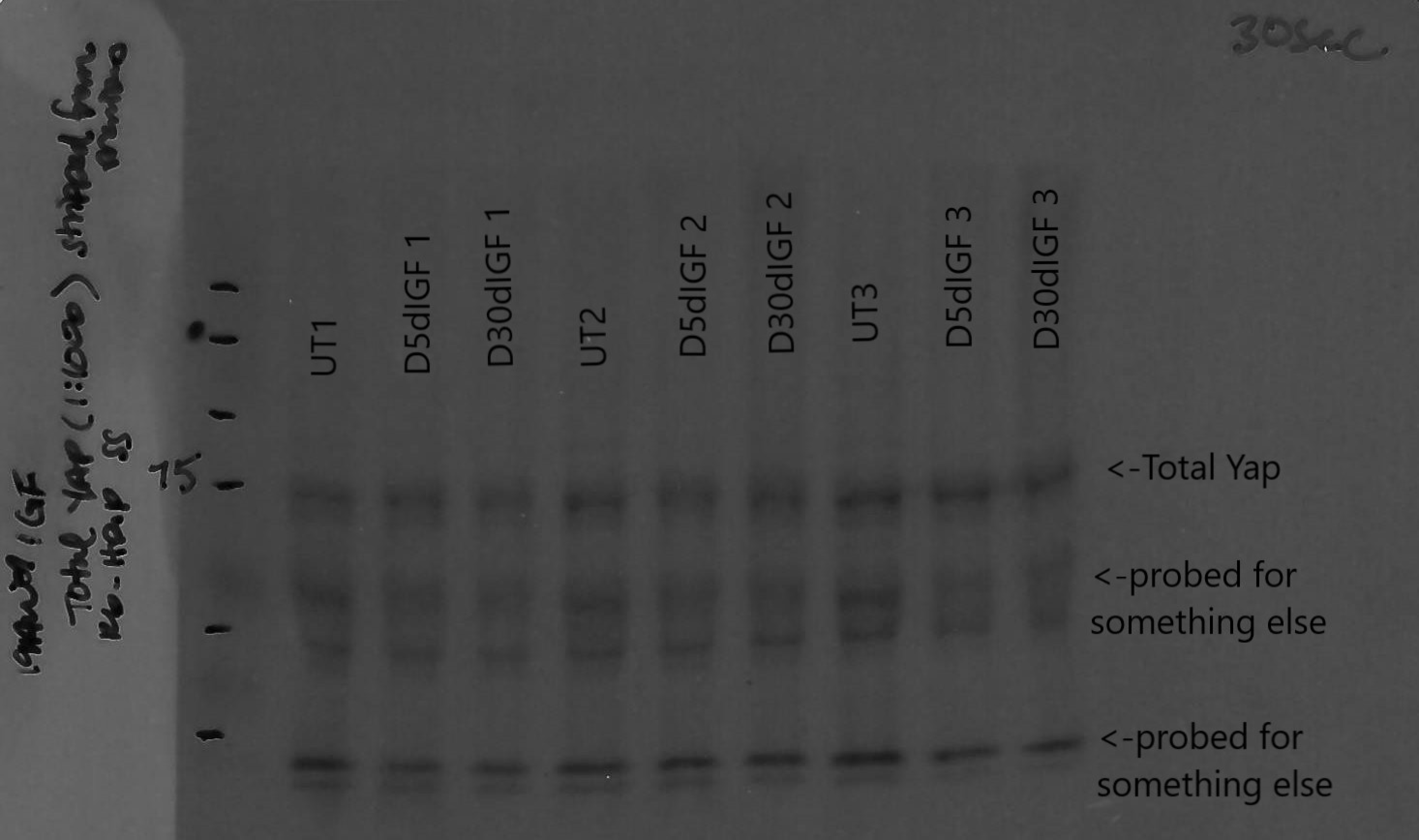
30sec
75
UT1
D5dIGF 1
D30dIGF 1
UT2
D5dIGF 2
D30dIGF 2
UT3
D5dIGF 3
D30dIGF 3
<-Total Yap
<-probed for something else
<-probed for something else

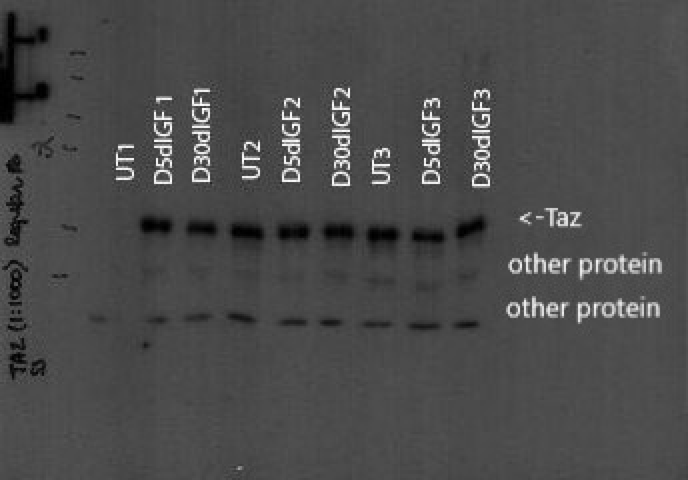
UT1
D5dIGF 1
D30dIGF1
UT2
D5dIGF2
D30dIGF2
UT3
D5dIGF3
D30dIGF3
<-Taz
other protein
other protein
TAZ (1:1000)

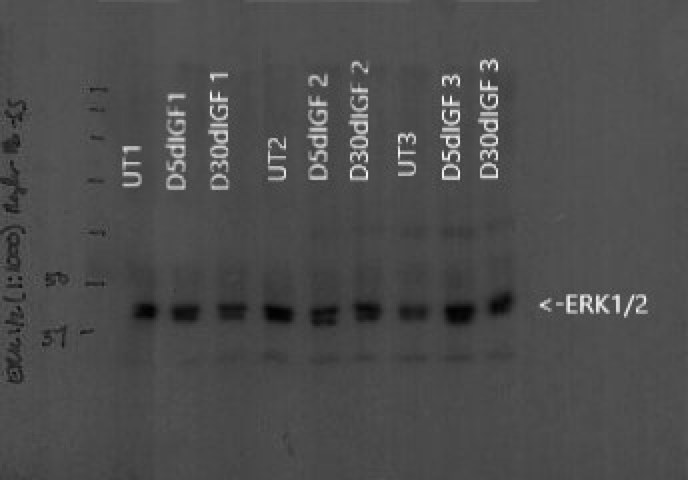
UT1
D5dIGF1
D30dIGF 1
UT2
D5dIGF 2
D30dIGF 2
UT3
D5dIGF 3
D30dIGF 3
<-ERK1/2

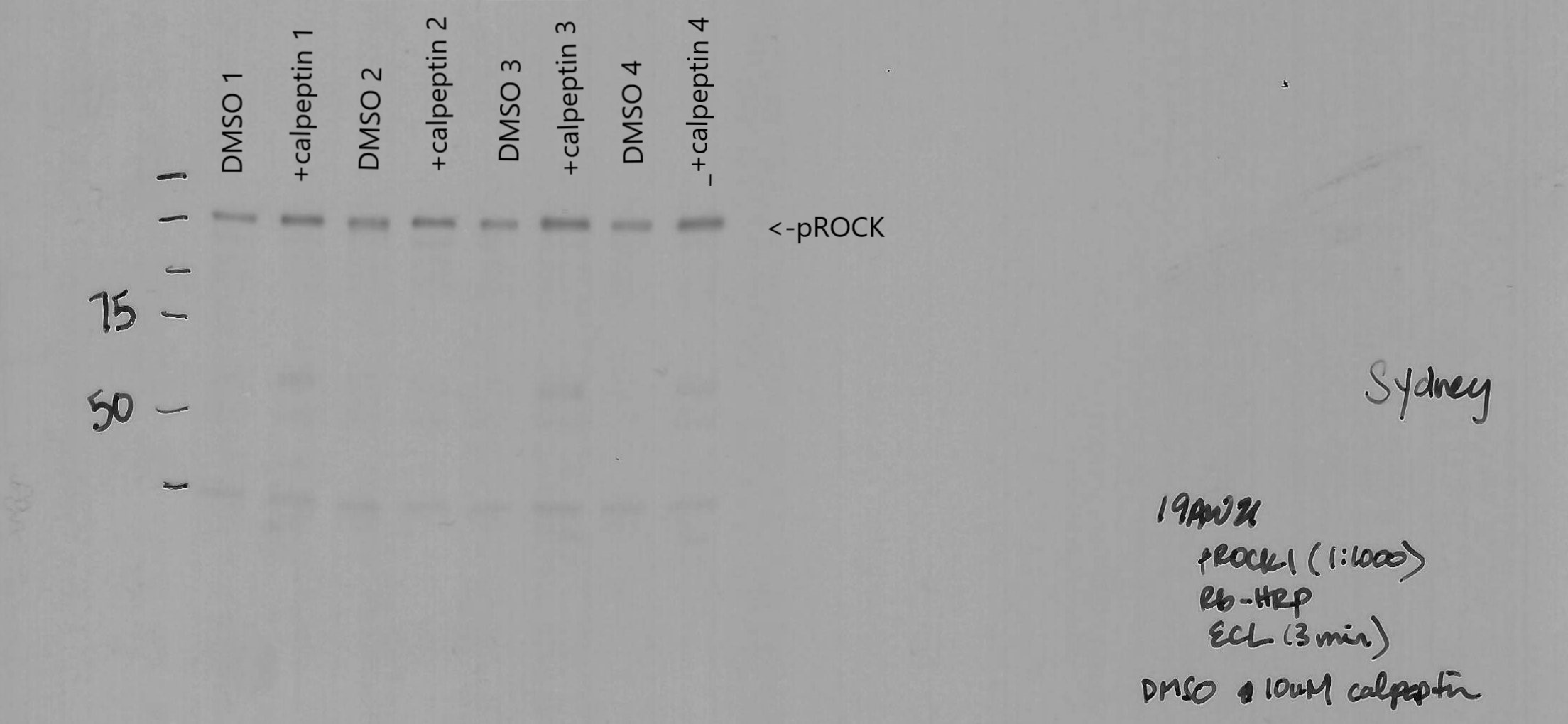
DMSO 1
+calpeptin 1
DMSO 2
+calpeptin 2
DMSO 3
+calpeptin 3
DMSO 4
+calpeptin 4
<-pROCK
75
50
Sydney
19AUG24
pROCK1 (1:1000)
Rb-HRP
ECL (3 min)
DMSO ± 10uM calpeptin

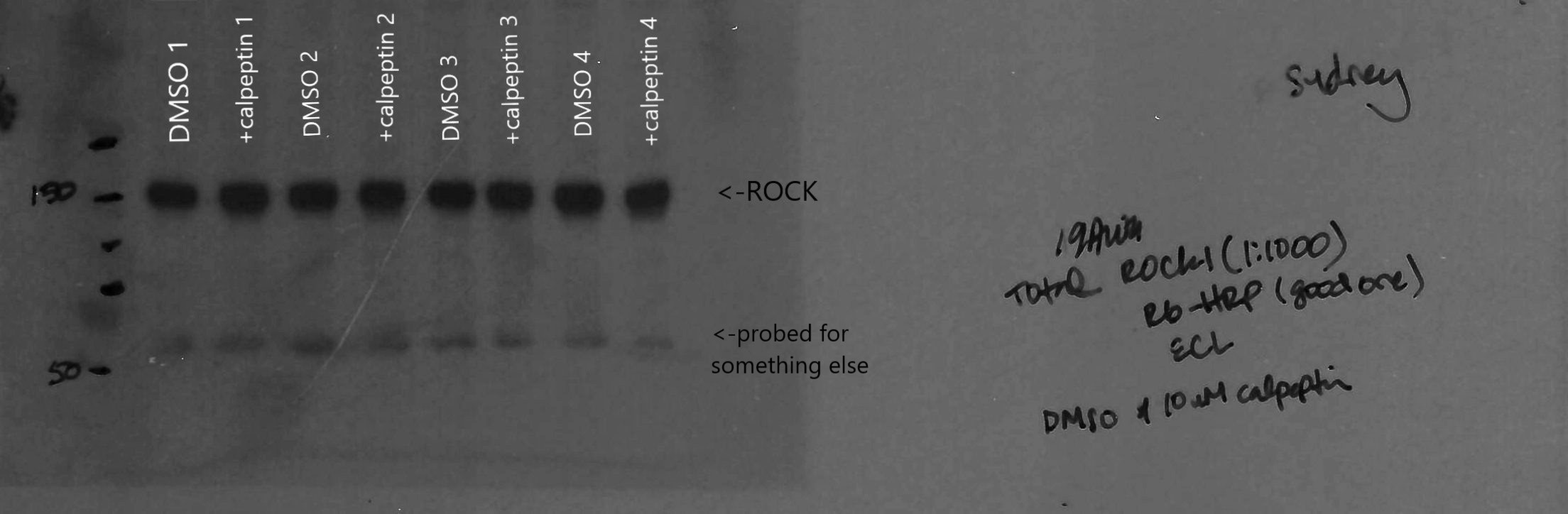

DMSO 1
+calpeptin 1
DMSO 2
+calpeptin 2
DMSO 3
+calpeptin 3
DMSO 4
+calpeptin 4
150
50
<-ROCK
<-probed for
something else
Sydney
Total ROCK1 (1:1000)
Rb-HRP (good one)
ECL
DMSO + 10 uM calpeptin

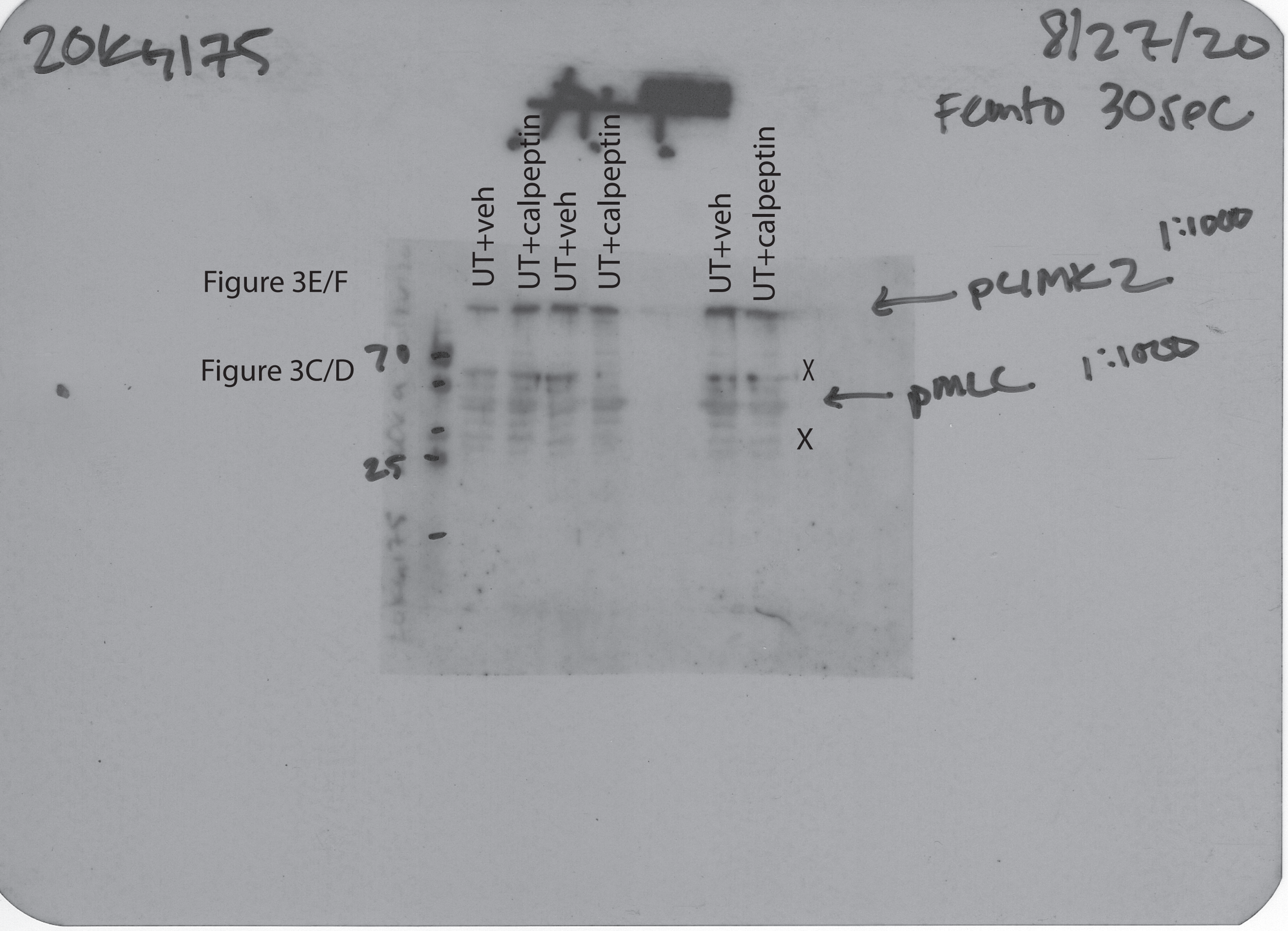
8/27/20
Femto 30sec
Figure 3E/F
Figure 3C/D
UT+veh
UT+calpeptin
UT+veh
UT+calpeptin
UT+veh
UT+calpeptin
70
25
X
X
1:1000
1:1000

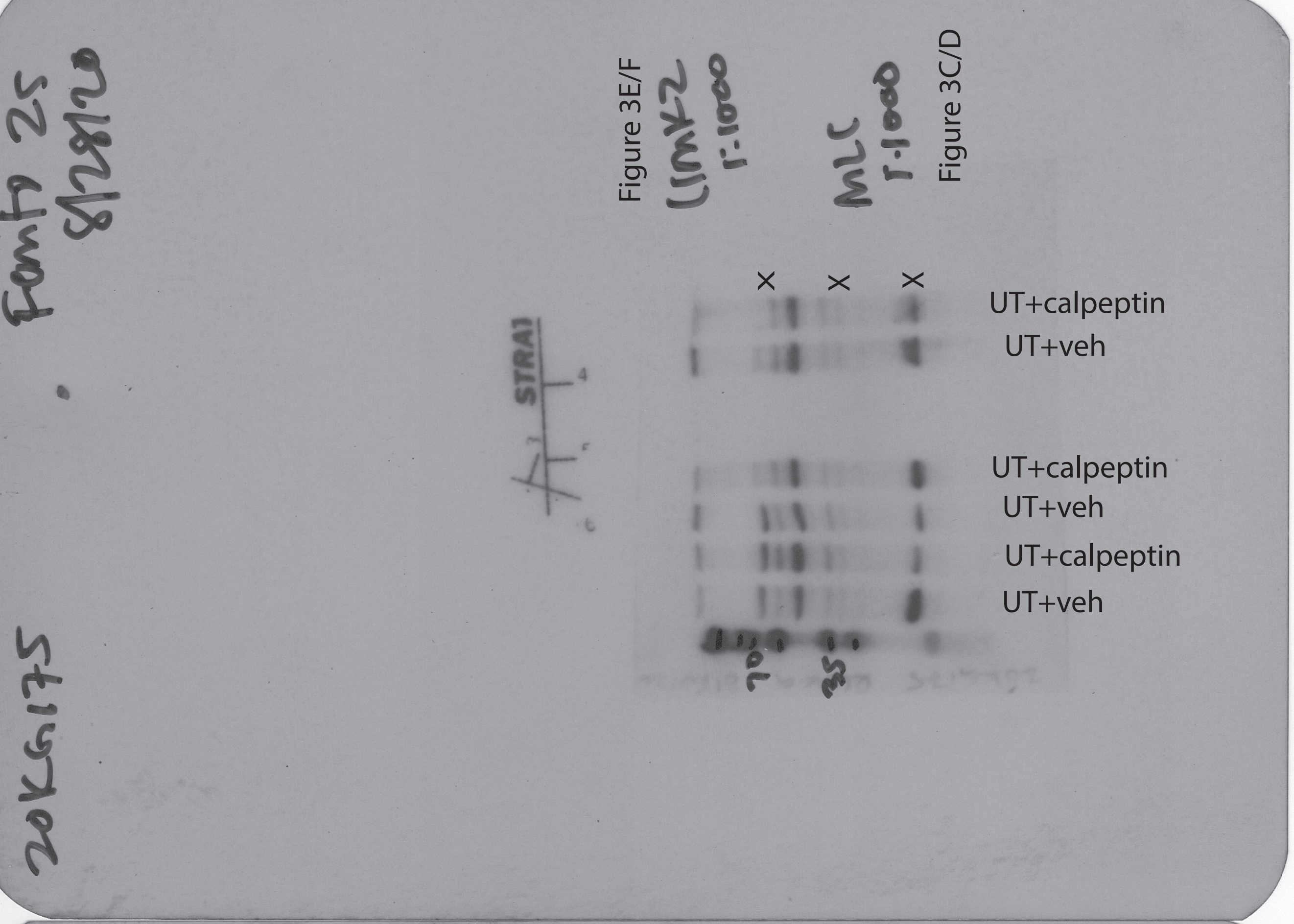
Figure 3E/F
LIMK2
1:1000
MLC
1:1000
Figure 3C/D
X
X
X
UT+calpeptin
UT+veh
UT+calpeptin
UT+veh
UT+calpeptin
UT+veh
70
35

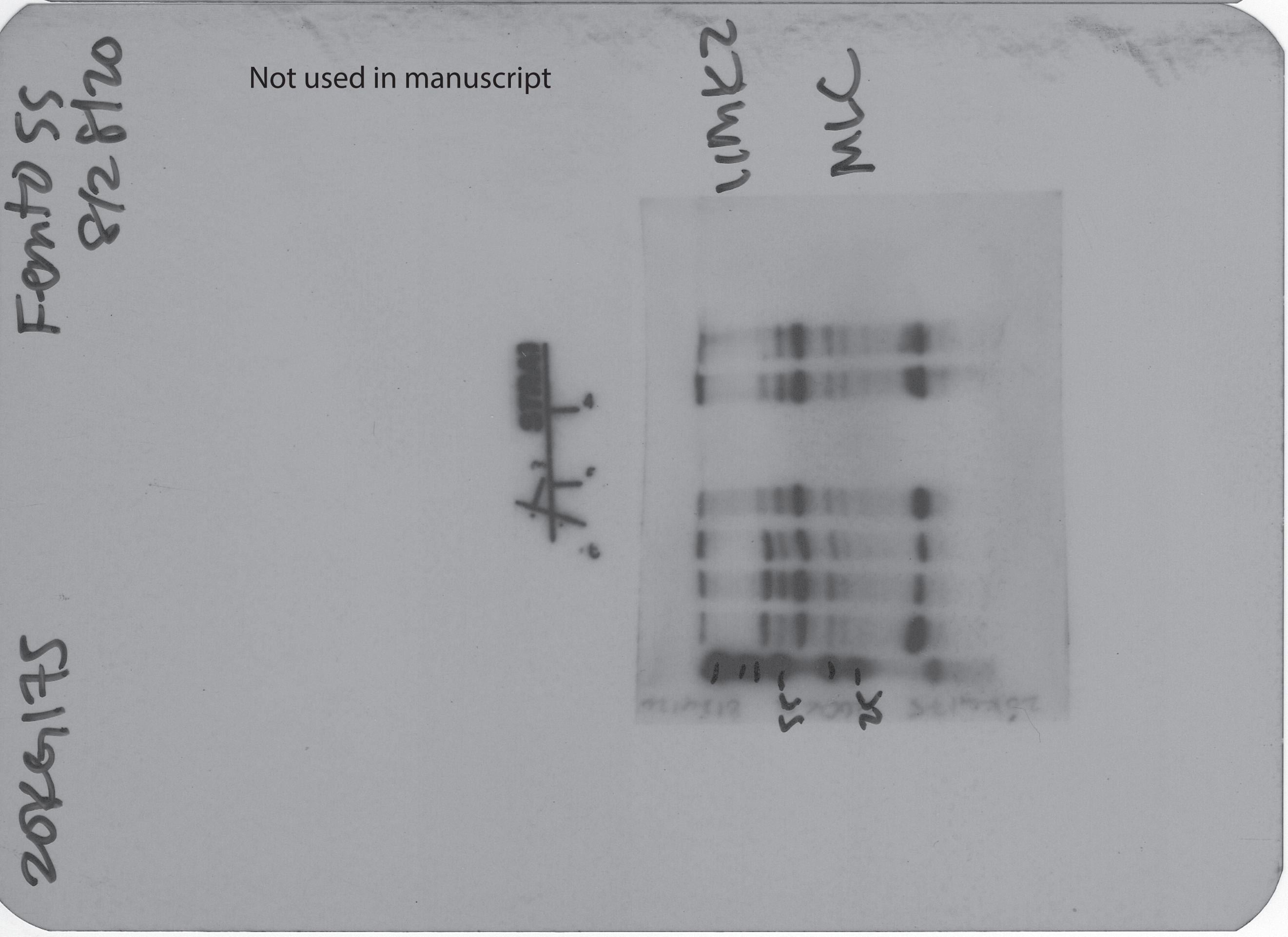
Not used in manuscript
LIMK2
MLC
55
25

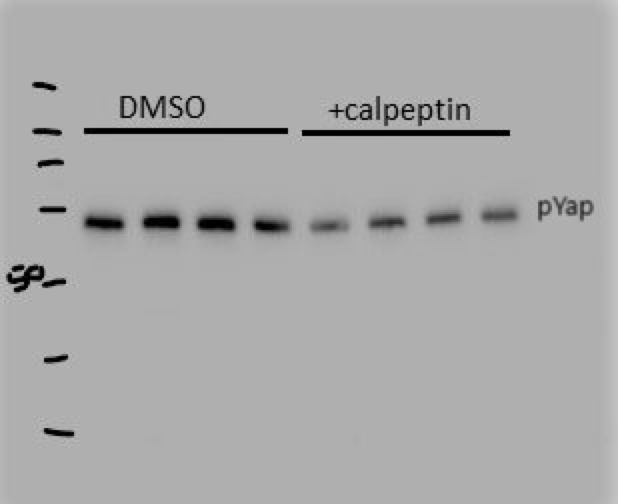
DMSO
+calpeptin
pYap

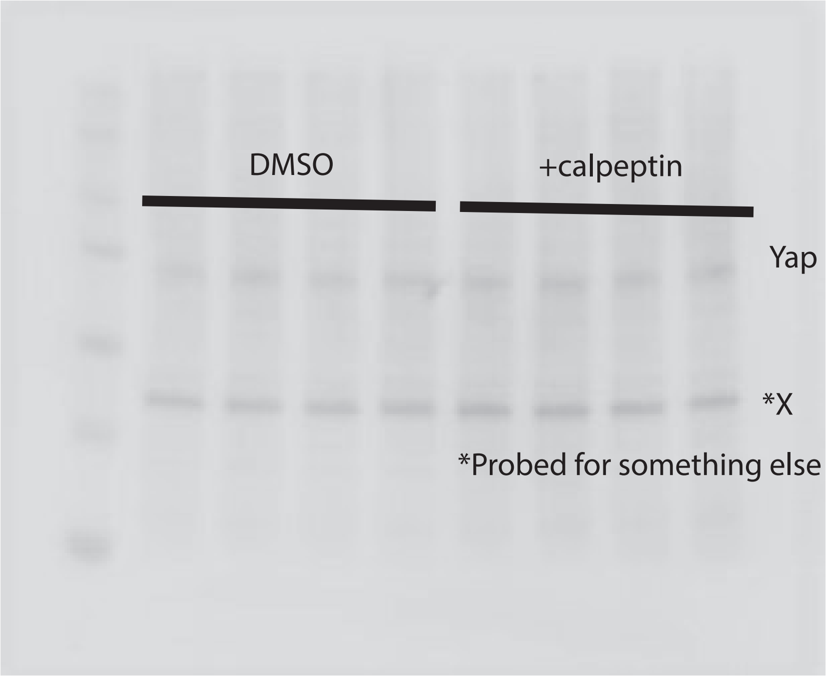
DMSO
+calpeptin
Yap
*X
*Probed for something else

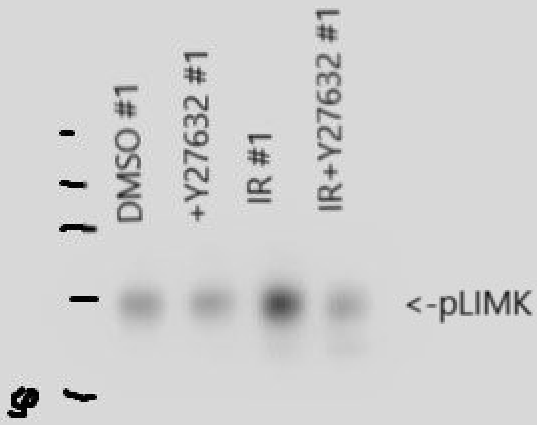

DMSO #1
+Y27632 #1
IR #1
IR+Y27632 #1
<-pLIMK

02/26/20
SSFemto 4min

Figure 4C

UT+vehicle
UT+ Y27632
5Gy+vehicle
5Gy+Y27632

Samples reanalyzed to confirm values
but not used for figures

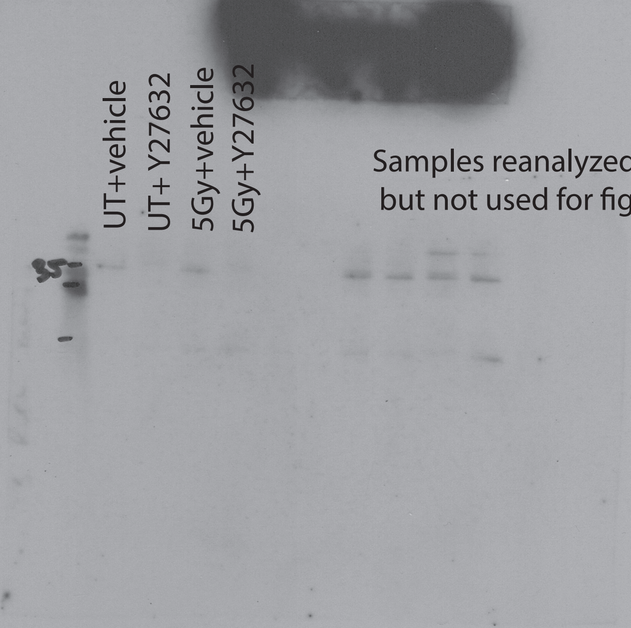

pMLC
1:1000

20KG173

Not used for manuscript

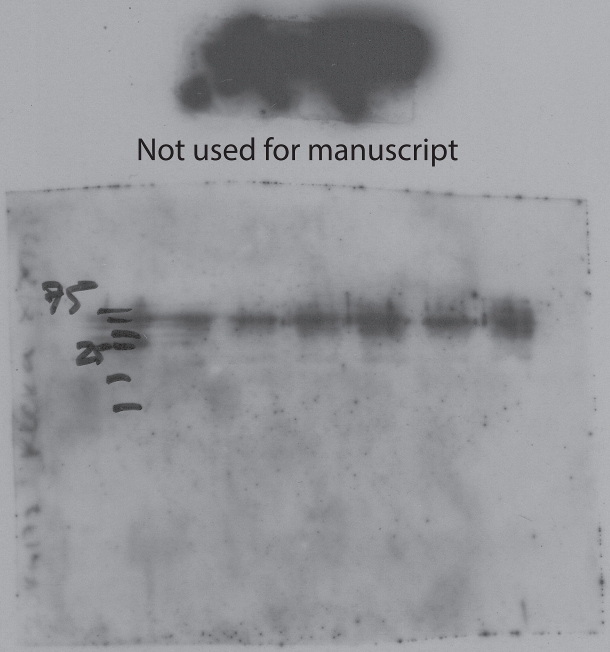

pLIMK2
1:1000

pMLC
1:1000

20KG172

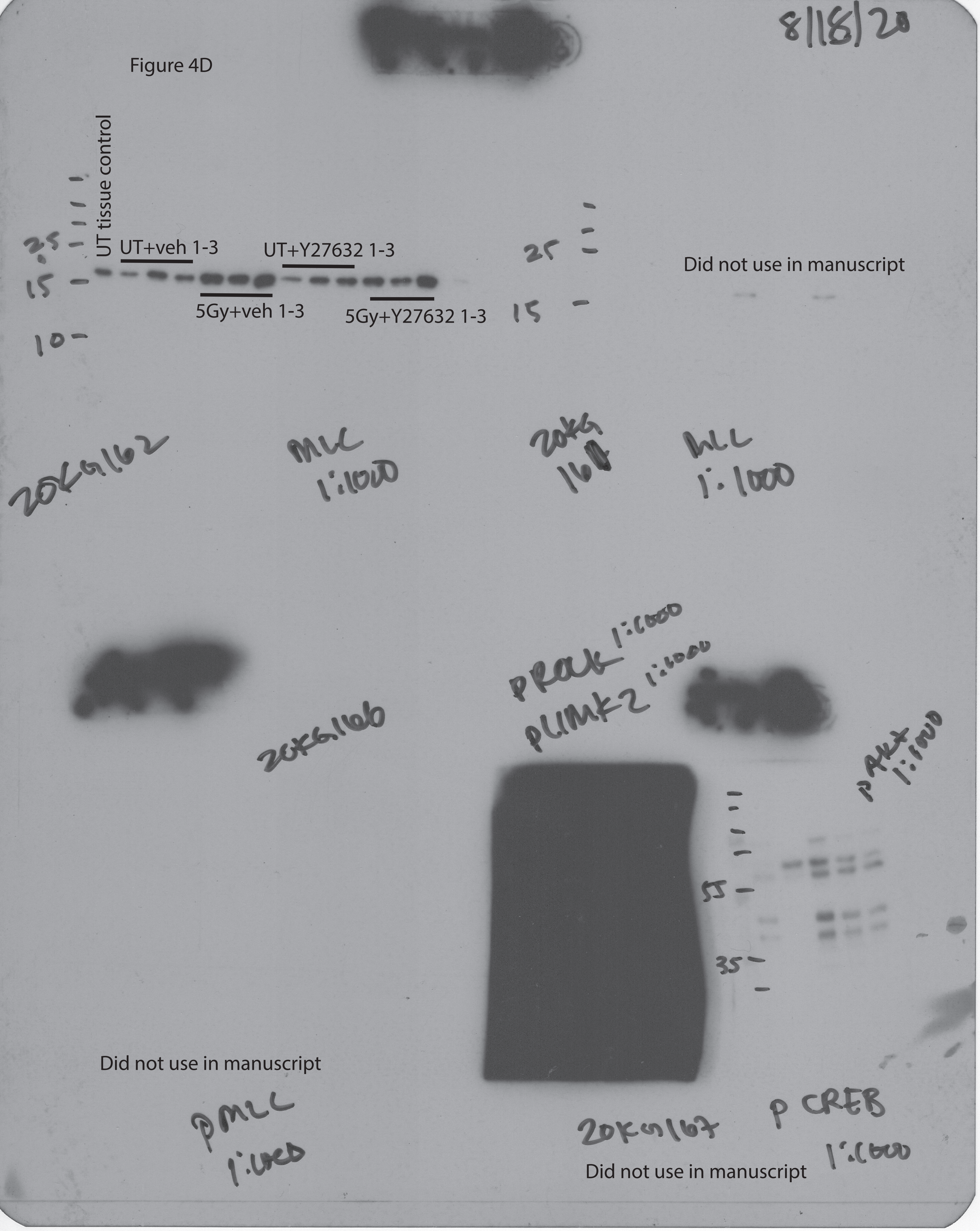

8/18/20
Figure 4D
UT tissue control
UT+veh 1-3
UT+Y27632 1-3
5Gy+veh 1-3
5Gy+Y27632 1-3
25
15
10
25
15
Did not use in manuscript
20KG162
MLC 1:1000
20KG 164
MLC 1:1000
pROCK 1:1000
pLIMK2 1:1000
20KG166
pAKT 1:1000
55
35
Did not use in manuscript
pMLC 1:1000
20KG167
pCREB 1:1000
Did not use in manuscript

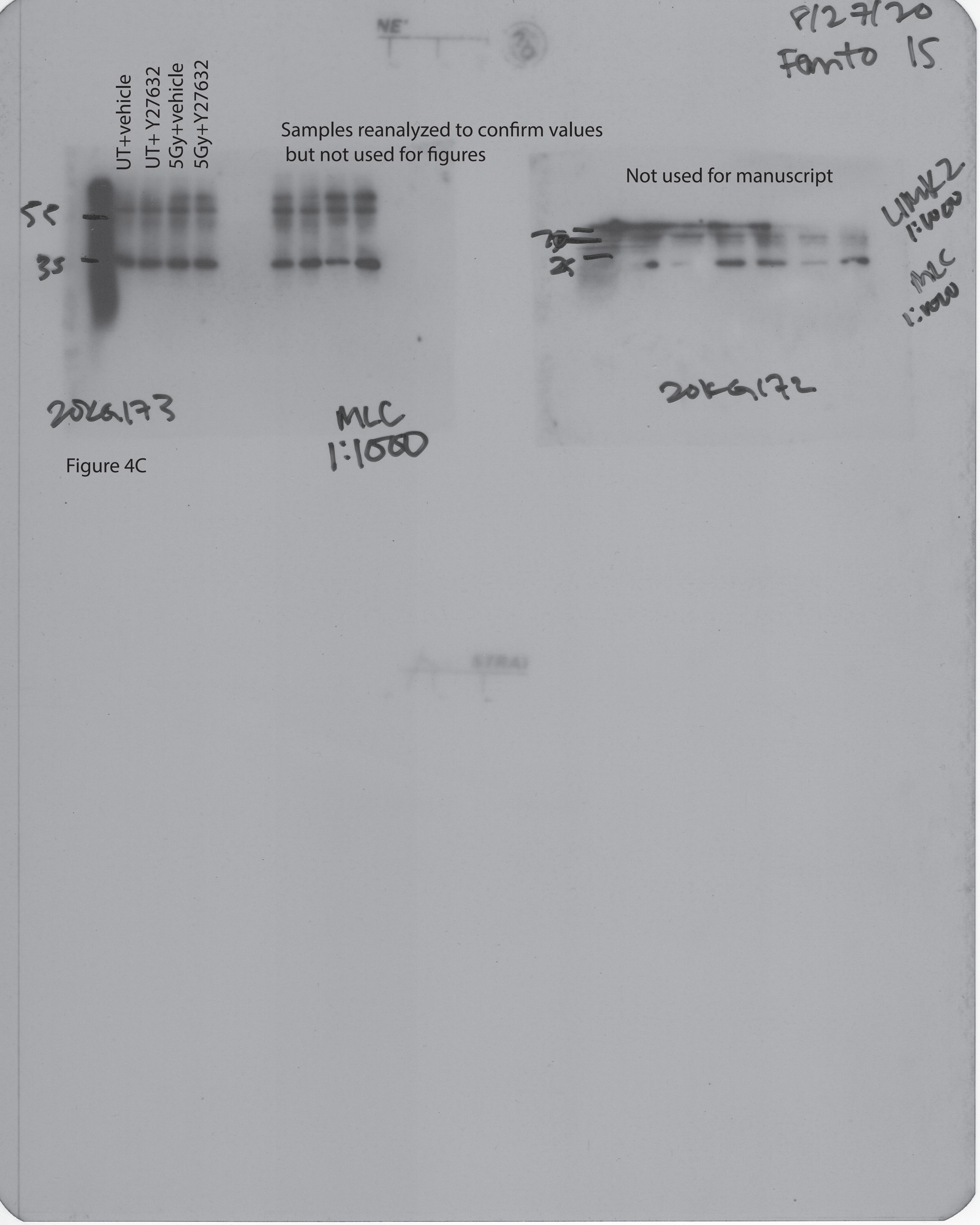

8/27/20
Femto 15
UT+vehicle
UT+ Y27632
5Gy+vehicle
5Gy+Y27632
Samples reanalyzed to confirm values but not used for figures
Not used for manuscript
55
35
70
25
LIMK2 1:1000
MLC 1:1000
20KG173
MLC 1:1000
20KG172
Figure 4C

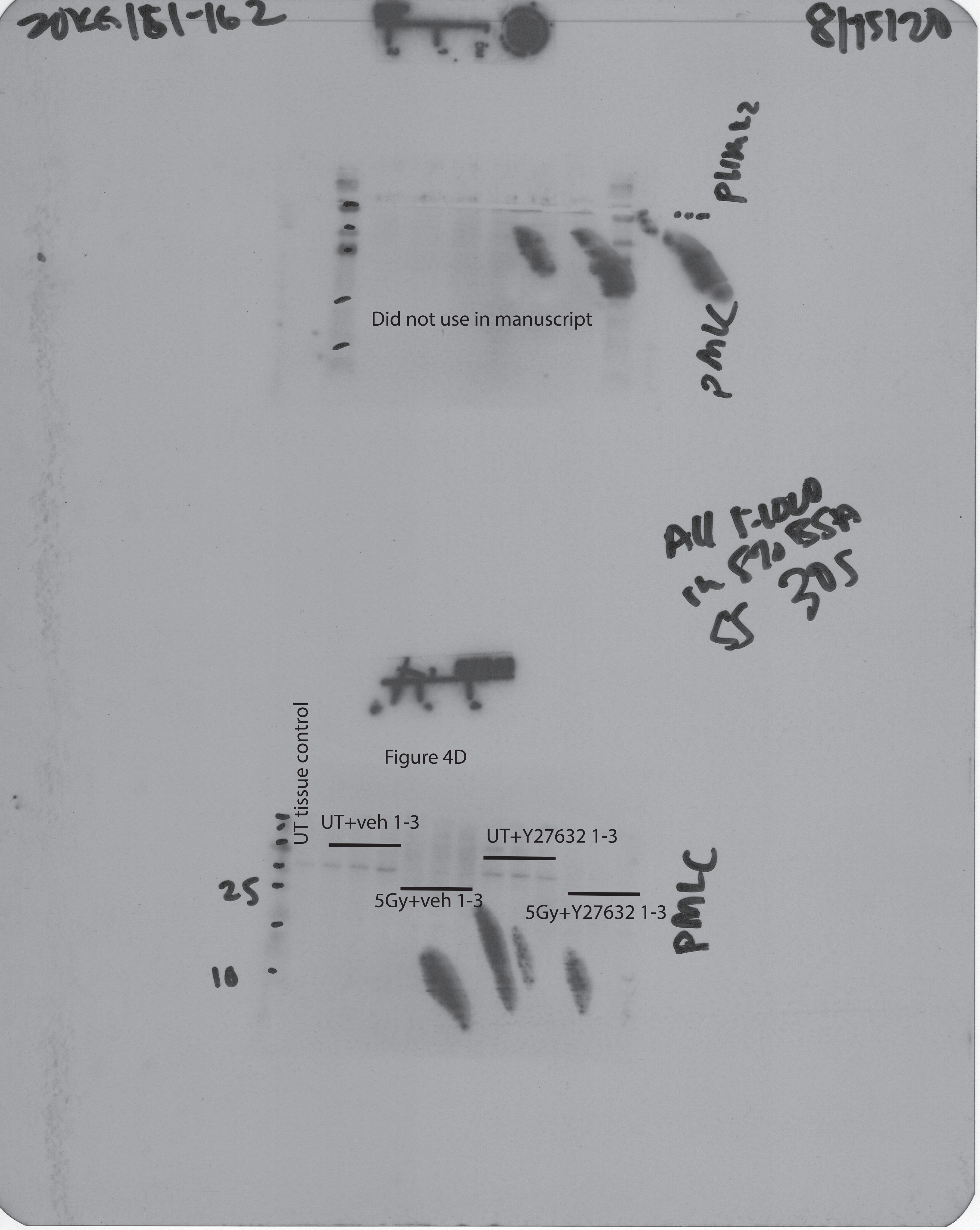

Did not use in manuscript
PMLC
Figure 4D
UT tissue control
UT+veh 1-3
UT+Y27632 1-3
5Gy+veh 1-3
5Gy+Y27632 1-3
25
10
PMLC

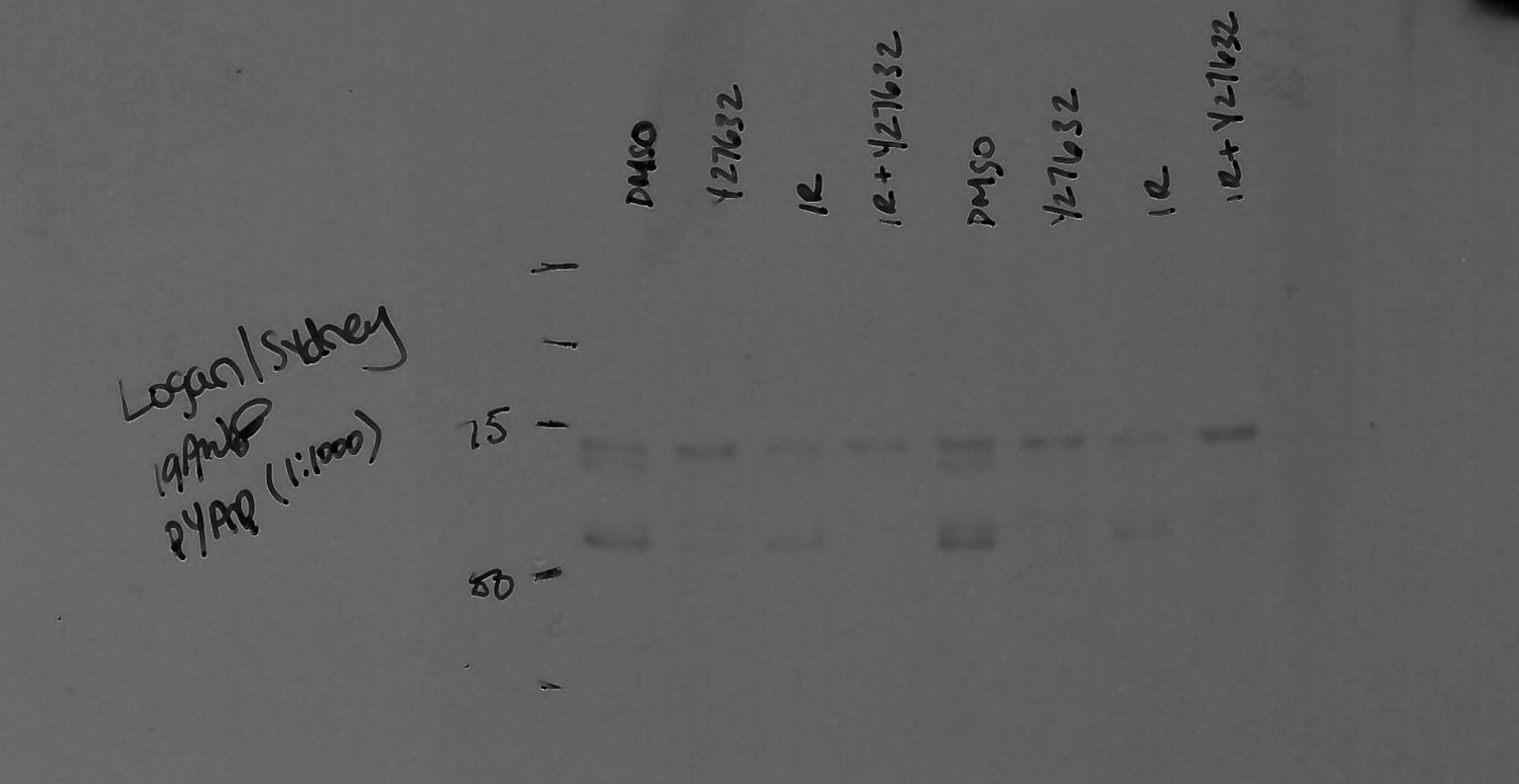
DMSO
Y27632
IR
IR + Y27632
DMSO
Y27632
IR
IR + Y27632
75
50
Logan/Sydney
pYAP (1:1000)

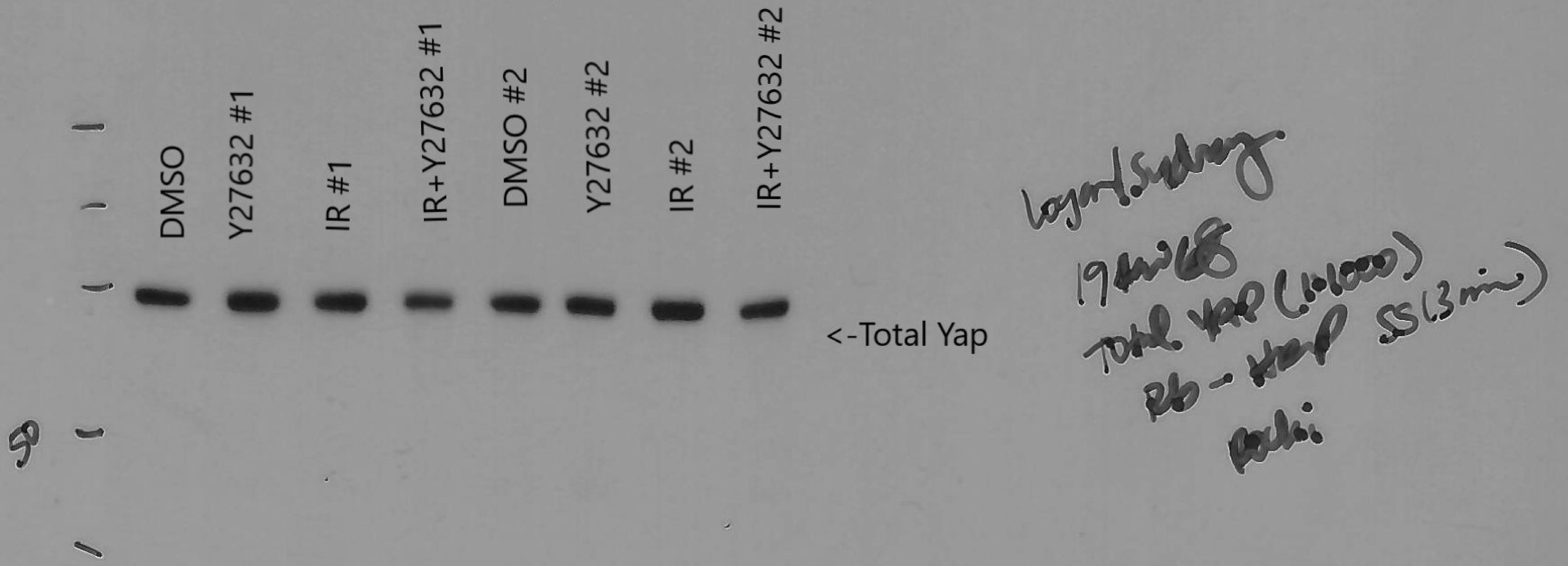
DMSO
Y27632 #1
IR #1
IR+Y27632 #1
DMSO #2
Y27632 #2
IR #2
IR+Y27632 #2
<-Total Yap
50
